# A Randomised Controlled Trial of Artemether-Lumefantrine Versus Artesunate for Uncomplicated Plasmodium falciparum Treatment in Pregnancy

**DOI:** 10.1371/journal.pmed.0050253

**Published:** 2008-12-23

**Authors:** Rose McGready, Saw Oo Tan, Elizabeth A Ashley, Mupawjay Pimanpanarak, Jacher Viladpai-nguen, Lucy Phaiphun, Katja Wüstefeld, Marion Barends, Natthapon Laochan, Lily Keereecharoen, Niklas Lindegardh, Pratap Singhasivanon, Nicholas J White, François Nosten

**Affiliations:** 1 Shoklo Malaria Research Unit (SMRU), Mae Sot, Tak, Thailand; 2 Faculty of Tropical Medicine, Mahidol University, Bangkok, Thailand; 3 Centre for Clinical Vaccinology and Tropical Medicine, Churchill Hospital, Oxford, United Kingdom; Royal Melbourne Hospital, Australia

## Abstract

**Background:**

To date no comparative trials have been done, to our knowledge, of fixed-dose artemisinin combination therapies (ACTs) for the treatment of Plasmodium falciparum malaria in pregnancy. Evidence on the safety and efficacy of ACTs in pregnancy is needed as these drugs are being used increasingly throughout the malaria-affected world. The objective of this study was to compare the efficacy, tolerability, and safety of artemether-lumefantrine, the most widely used fixed ACT, with 7 d artesunate monotherapy in the second and third trimesters of pregnancy.

**Methods and Findings:**

An open-label randomised controlled trial comparing directly observed treatment with artemether-lumefantrine 3 d (AL) or artesunate monotherapy 7 d (AS7) was conducted in Karen women in the border area of northwestern Thailand who had uncomplicated P. falciparum malaria in the second and third trimesters of pregnancy. The primary endpoint was efficacy defined as the P. falciparum PCR-adjusted cure rates assessed at delivery or by day 42 if this occurred later than delivery, as estimated by Kaplan-Meier survival analysis. Infants were assessed at birth and followed until 1 y of life. Blood sampling was performed to characterise the pharmacokinetics of lumefantrine in pregnancy. Both regimens were very well tolerated. The cure rates (95% confidence interval) for the intention to treat (ITT) population were: AS7 89.2% (82.3%–96.1%) and AL 82.0% (74.8%–89.3%), *p* = 0.054 (ITT); and AS7 89.7% (82.6%–96.8%) and AL 81.2% (73.6%–88.8%), *p* = 0.031 (per-protocol population). One-third of the PCR-confirmed recrudescent cases occurred after 42 d of follow-up. Birth outcomes and infant (up to age 1 y) outcomes did not differ significantly between the two groups. The pharmacokinetic study indicated that low concentrations of artemether and lumefantrine were the main contributors to the poor efficacy of AL.

**Conclusion:**

The current standard six-dose artemether-lumefantrine regimen was well tolerated and safe in pregnant Karen women with uncomplicated falciparum malaria, but efficacy was inferior to 7 d artesunate monotherapy and was unsatisfactory for general deployment in this geographic area. Reduced efficacy probably results from low drug concentrations in later pregnancy. A longer or more frequent AL dose regimen may be needed to treat pregnant women effectively and should now be evaluated. Parasitological endpoints in clinical trials of any antimalarial drug treatment in pregnancy should be extended to delivery or day 42 if it comes later.

**Trial Registration:** Current Controlled Trials ISRCTN86353884

## Abstract 2

Rose McGready and colleagues show that an artemether-lumefantrine regimen is well tolerated and safe in pregnant Karen women with uncomplicated falciparum malaria, but efficacy is inferior to artesunate, probably because of low drug concentrations in later pregnancy.

## Editors' Summary

**Background.:**

Plasmodium falciparum, a mosquito-borne parasite that causes malaria, kills nearly one million people every year. Although most deaths occur among young children, malaria during pregnancy is also an important public-health problem. In areas where malaria transmission is high (stable transmission), women acquire a degree of immunity. Although less symptomatic than women who lack natural protection, their babies are often small and sickly because malaria-related anemia (lack of red blood cells) and parasites in the placenta limit the nutrients supplied to the baby before birth. By contrast, in areas where malaria transmission is low (unstable transmission or sporadic outbreaks), women have little immunity to P. falciparum. If these women become infected during pregnancy, “uncomplicated” malaria (fever, chills, and anemia) can rapidly progress to “severe” malaria (in which vital organs are damaged), which can be fatal to the mother and/or her unborn child unless prompt and effective treatment is given.

**Why Was This Study Done?:**

Malaria parasites are now resistant to many of the older antimalarial drugs (for example, quinine). So, since 2006, the World Health Organization (WHO) has recommended that uncomplicated malaria during the second and third trimester of pregnancy is treated with short course (3 d) fixed-dose artemisinin combination therapy (ACT; quinine is still used in early pregnancy because it is not known whether ACT damages fetal development, which mainly occurs during the first 3 mo of pregnancy). Artemisinin derivatives are fast-acting antimalarial agents that are used in combination with another antimalarial drug to reduce the chances of P. falciparum becoming resistant to either drug. The most widely used fixed-dose ACT is artemether–lumefantrine (AL) but, although several trials have examined the safety and efficacy of this treatment in non-pregnant women, little is known about how well it works in pregnant women. In this study, the researchers compare the efficacy, tolerability, and safety of AL with a 7-d course of artesunate monotherapy (AS7; another artemisinin derivative) in the treatment of uncomplicated malaria in pregnancy in northwest Thailand, an area with unstable but highly drug resistant malaria transmission.

**What Did the Researchers Do and Find?:**

The researchers enrolled 253 women with uncomplicated malaria during the second and third trimesters of pregnancy into their open-label trial (a trial in which the patients and their health-care workers know who is receiving which drug regimen). Half the women received each type of treatment. The trial's main outcome was the “PCR-adjusted cure rate” at delivery or 42 d after treatment if this occurred after delivery. This cure rate was assessed by examining blood smears for parasites and then using a technique called PCR to determine which cases of malaria were new infections (classified as treatment successes along with negative blood smears) and which were recurrences of an old infection (classified as treatment failures). The PCR-adjusted cure rates were 89.7% and 81.2% for AS7 and AL, respectively. Both treatments were well tolerated, few side effects were seen with either treatment, and infant health and development at birth and up to 1 y old were similar with both regimens. Finally, an analysis of blood samples taken 7 d after treatment with AL showed that blood levels of lumefantrine were below those previously associated with treatment failure in about a third of the women tested.

**What Do These Findings Mean?:**

Although these findings indicate that the AL regimen is a well tolerated and safe treatment for uncomplicated malaria in pregnant women living in northwest Thailand, the efficacy of this treatment was lower than that of artesunate monotherapy. In fact, neither treatment reached the 90% cure rate recommended by WHO for ACTs and it is likely that cure rates in a more realistic situation (that is, not in a trial where efforts are made to make sure everyone completes their treatment) would be even lower. The findings also suggest that the reduced efficacy of the AL regimen in pregnant women compared to the efficacy previously seen in non-pregnant women may be caused by lower drug blood levels during pregnancy. Thus, a higher-dose AL regimen (or an alternative ACT) may be needed to successfully treat uncomplicated malaria during pregnancy.

**Additional Information.:**

Please access these Web sites via the online version of this summary at http://dx.doi.org/10.1371/journal.pmed.0050253.

- The MedlinePlus encyclopedia contains a page on malaria (in English and Spanish)
- Information is available from the World Health Organization on malaria (in several languages), and their 2006 Guidelines for the Treatment of Malaria includes specific recommendations for the treatment of pregnant women
- The US Centers for Disease Control and Prevention provide information on malaria and on malaria during pregnancy (in English and Spanish)
- Information is available from the Roll Back Malaria Partnership on malaria during pregnancy, on artemisinin-based combination therapies, and on malaria in Thailand

## Introduction

Plasmodium falciparum infections in pregnancy account for a high proportion of maternal mortality in malaria-endemic countries [1,2], particularly in areas of low and unstable transmission. The northwestern border of Thailand is an area of low seasonal malaria transmission complicated by high levels of multidrug resistance in P. falciparum. In this region in 1986 an estimated 1% of all pregnant women died from malaria annually. Since then there has been no safe and effective chemoprophylaxis that could be offered to pregnant women. A system of weekly antenatal clinics (ANCs) was therefore started. The ANCs provided early detection and treatment of malaria and reduced both morbidity and mortality [3]. Other malaria-preventive approaches in pregnancy such as insecticide-treated nets [4] and insect repellents applied to the skin [5] did not show a significant preventive effect in this area. The therapeutic responses to various antimalarial drug treatments of multidrug-resistant falciparum malaria in pregnancy have been disappointing. In this geographic area, quinine currently cures less than 60% of pregnant patients [6,7]. This low cure rate is improved by combining quinine with clindamycin, but the poor tolerability of quinine and the consequent poor adherence to 7 d treatment regimens compromise effectiveness [8]. Short course (3 d) fixed-dose artemisinin combination therapies (ACTs) are now recommended by the World Health Organization as first-line treatments of falciparum malaria in all endemic areas, and endorsed for use in the second and third trimesters of pregnancy [9].

Artemether-lumefantrine given twice daily for 3 d (AL), is the most widely used fixed-dose ACT. It is effective against multidrug-resistant P. falciparum malaria [10,11] but there are no published efficacy data in pregnancy [9]. AL has an excellent safety profile in non-pregnant humans [12]. In animal studies, lumefantrine was neither mutagenic nor embryotoxic [13]. Artemether, like all artemisinin derivatives, can induce foetal resorption if given in high doses to experimental animals during a narrow time window in early gestation [14,15], although recent studies in primates showed no teratogenic effects at dosages equivalent to those currently recommended for antimalarial treatment [16].

In 1994 the northwestern border of Thailand became one of the first areas in which ACTs were deployed at scale. It took until 2003 for sufficient experience with artesunate treatment in pregnancy to accumulate [17–19], and for sufficient confidence to develop for ethical approval to be granted for a treatment protocol evaluating AL in pregnancy. In 2006 WHO published guidelines recommending ACTs for the treatment of malaria in the second and third trimester of pregnancy whilst acknowledging the urgent need for more information, on both safety and dosing [9]. There is increasing evidence that the pharmacokinetic properties of several antimalarial drugs, including artemisinin derivatives, are affected significantly in late pregnancy resulting in lower blood concentrations. In the study area, current first-line treatment for falciparum malaria in pregnancy is quinine and clindamycin in the first trimester, and artesunate and clindamycin in the second and third trimesters. These are all 7-d treatments. A safe and effective shorter-course fixed dose ACT is needed to improve adherence to treatment and ensure high cure rates in pregnancy. However there are concerns that currently recommended doses of antimalarials in pregnant women may be suboptimal [20–22]. The hypothesis this study tested was that the AL would be superior to artesunate monotherapy 7 d (AS7) in terms of parasitological efficacy in the treatment of uncomplicated P. falciparum malaria in the second and third trimesters of pregnancy. This randomised comparative trial included a recently published nested substudy in which the pharmacokinetic properties of artemether, dihydroartemisinin, and lumefantrine [23] were characterised, followed by larger evaluations of lumefantrine population pharmacokinetics and day 7 drug level monitoring.

## Materials and Methods

The CONSORT checklist and trial protocol can be found in Text S1 and S2, respectively.

### Participants Setting and Location

The women in this study attended the weekly ANC of Shoklo Malaria Research Unit (SMRU). ANCs have been conducted by the SMRU in Maela refugee camp since 1995, for migrants south of Mae Sot since 1998, and for migrants north of Mae Sot since 2004. These ANCs are attended by women from approximately 120 km of the border area between Thailand and Burma, north and south of the border town of Mae Sot.

**Figure 1:**
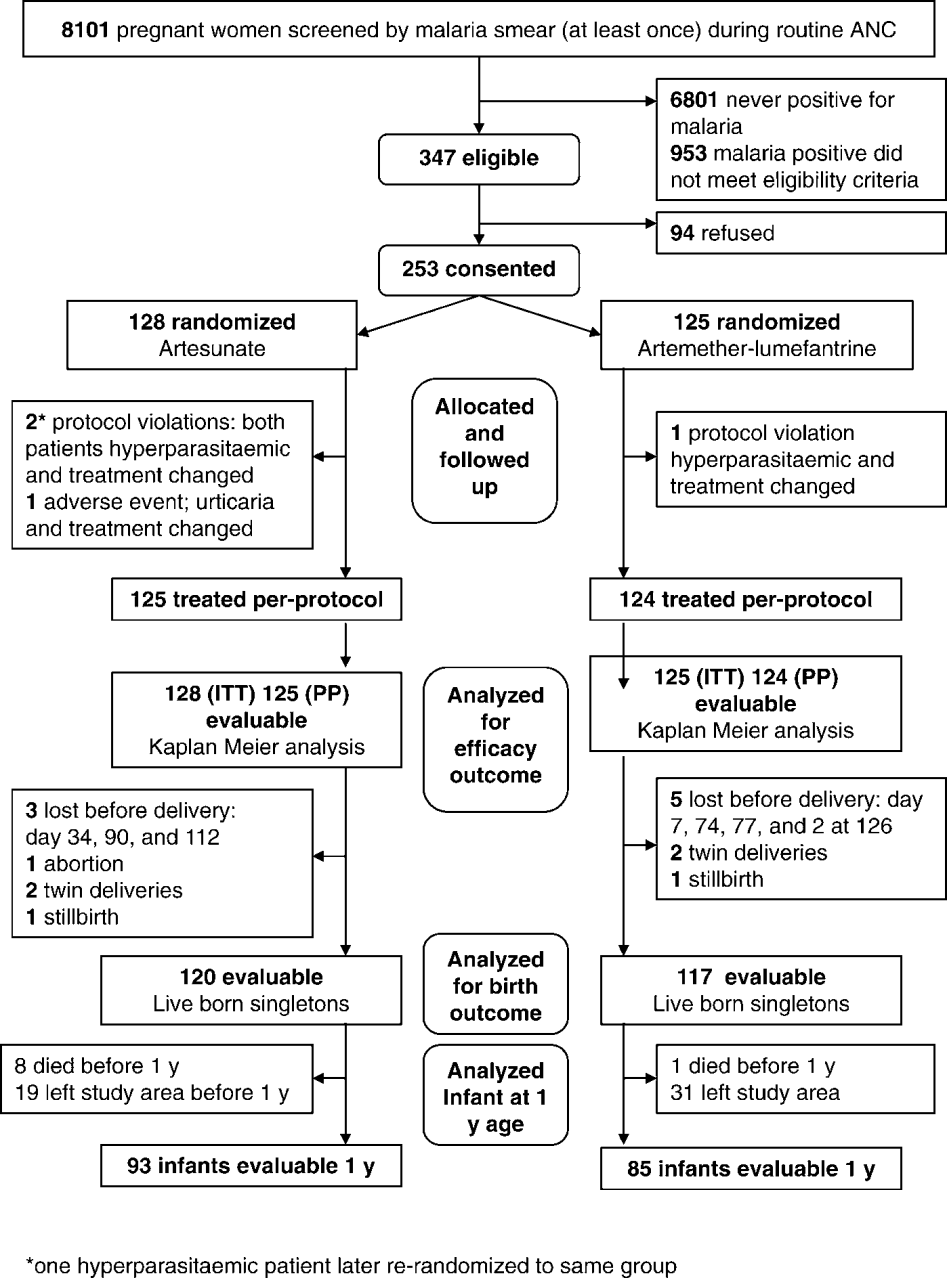
Participant Flow in the Randomised Comparison of AS7 and AL

**Figure 2:**
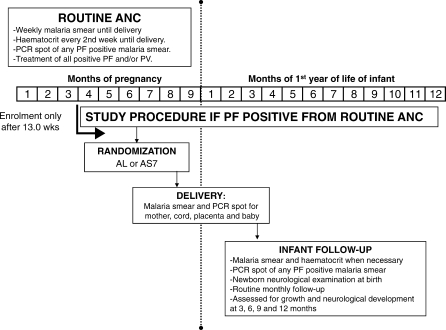
Schematic Diagram of Routine Antenatal Care and Basic Study Procedures in Pregnancy, at Delivery and Infant Follow-up

A blood smear to detect malaria is taken at each weekly ANC visit (Figure 1). All parasitaemic episodes during pregnancy are treated (Figure 2). In addition, women positive for P. falciparum have blood spots taken for PCR genotyping so that treatment efficacy can be assessed accurately (Figure 2). The haematocrit is measured every 2 wk (Figure 2). Women receive weekly ferrous sulphate (200 mg daily) and folic acid (5 mg once weekly) prophylaxis or treatment for anaemia, from the first ANC consultation until delivery. All medical and obstetric problems in pregnancy are investigated and treated free of charge, resulting in low rates of self-medication. All women are encouraged to deliver under supervision in the SMRU facilities. Complicated deliveries requiring cesarean section are referred to Mae Sot hospital (1 h drive).

### Eligibility

Patients with acute falciparum malaria in the second or third trimesters of pregnancy were studied (Figure 2). Patients with previous splenectomy, known chronic disease (cardiac, renal, hepatic, haemoglobinopathy), inability to follow the ANC consultation, history of alcohol or narcotic abuse, imminent delivery, hyperparasitaemic and severe malaria, inability to tolerate oral treatment, or allergy to artemisinin derivatives or lumefantrine, were excluded from the study. The aims and procedures of the study and the anticipated benefits, and potential inconveniences and hazards were explained to each of the patients in her own language. Witnessed written consent or thumbprint was obtained from every patient participating in the study.

One of the ethical committees preferred that the inclusion criteria only permit pregnant women in the second and third trimester with a second (i.e., presumed recrudescent) episode of uncomplicated P. falciparum malaria to participate in the trial initially (Figure 2). Following a Data Safety Monitoring Committee review of the first 20 deliveries, eligibility was widened to allow any malaria episode, including the first episode in pregnancy in the second or third trimester, to be included.

### Ethics

The proposal was approved by three independent bodies: The Ethical Committee of the Faculty of Tropical Medicine, Mahidol University in Bangkok, the Oxford Tropical Research Ethic Committee, and the Secretariat Committee on Research Involving Human Subjects of the World Health Organization. The protocol for this study was first submitted to ethical review boards in January 2003 and the first of three committees granted ethical approval on 7 February 2003. The last gave approval on 6 February 2004. At the time of protocol submission 7 d of quinine (first trimester) and 7 d of artesunate (second and third trimester) were used alone for first-line treatment of uncomplicated P. falciparum. The second-line treatment was a 7 d combination of artesunate and clindamycin. This explains why artesunate monotherapy was chosen as the comparator for AL.

### Interventions

#### Antimalarial drug treatments.

Patients were randomised to receive directly observed treatment with either (a) 3 d artemether-lumefantrine (Novartis, Basel, Switzerland, 20/120 mg artemether/lumefantrine): four tablets twice a day for 3 d, with 250 ml of chocolate milk containing 7 g of fat at each dose (the drug was given at 0, 8, 24, 36, 48 and 60 h), or (b) artesunate 2 mg/kg (50 mg tablets from Guilin Pharmaceutical Factory, Guangxi, PRC) once daily for 7 d.

All patients were admitted for the duration of the treatment and each antimalarial dose was supervised. The dose was repeated in full if vomiting occurred within 30 min after administration, or by a half dose if vomiting occurred between 30 and 60 min.

Two batches of AL were used during the course of the study. Drugs were stored mainly in the central pharmacy in Mae Sot with a smaller amount being made available for use in the field. All drugs were stored in a cool, dry place.

After the consent procedure and before drug administration a full medical history and examination (including obstetric evaluation) was carried out by a physician and recorded. This included recording of duration of symptoms before presentation and any drugs taken prior to arriving at the clinic.

**Figure 3:**
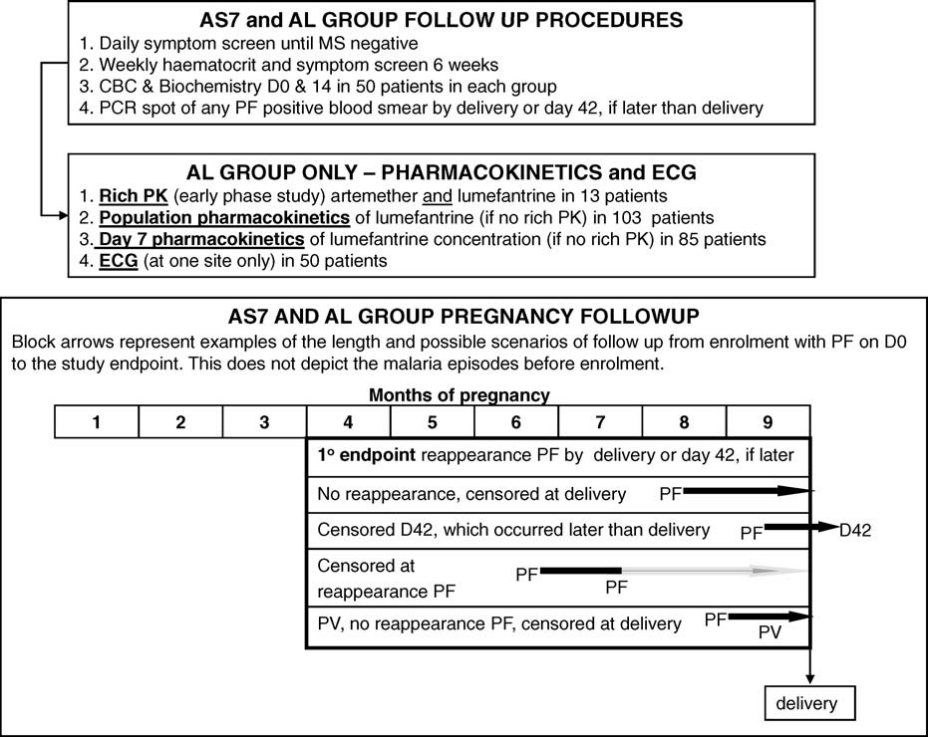
Schematic Diagram of Participant Follow-up after Randomization, Embedded Pharmacokinetic Studies, and Follow-up Scenarios D42, day 42; D0, day 0; PCR, polymerase chain reaction, PF, P. falciparum; PV, P. vivax.

Daily malaria smears were made until the patient was malaria smear–negative. Aural temperature was measured daily while the patient was hospitalised. Patients under treatment had daily clinical and obstetric examination, drug administration, and recording of adverse events (AEs) on a case record form (Figure 3). Thereafter patients were seen weekly until delivery or day 42 if this occurred after delivery (Figure 3). Reappearance of P. falciparum was treated with artesunate (2 mg/kg per day) and clindamycin (300 mg three times daily) for 7 d. Treatment of P. vivax infections was with a standard chloroquine regimen (25 mg base/kg over 3 d).

Labour was monitored using the WHO partogram and data on birth outcomes were collected. Low birth weight was defined as a birth weight measured in the first 72 h of life of < 2500 g and prematurity defined as delivery before 37.0 wk of gestation. Each baby was examined by a trained physician for the presence of congenital abnormalities and neurological development. Gestational age was estimated by ultrasound or by the Dubowitz examination [24] for those with a late ultrasound scan. All babies had a neonatal neurological examination [25]. The neurological optimality score was calculated for term singleton infants, born with cephalic presentation and examined within 72 h of birth [25]. Infants were followed monthly for growth assessment. Developmental assessments were carried out at 3, 6, 9, and 12 mo to determine the total developmental scores of the Shoklo Developmental test [26] . Attainment of motor milestones was evaluated at 12 mo (Figure 2).

AEs were defined as signs and symptoms that occurred or became more severe after treatment started. Symptoms were screened for at each visit for 6 wk. The causal relationship between the event and the study treatment was defined as unrelated, unlikely, possible, probable, or definite [27]. A subgroup of 50 patients in each group was asked to provide a complete blood count and biochemistry sample on admission and at day 14. In addition, 50 patients in the AL group had electrocardiographs (ECGs) recorded at baseline and 1 h after the last dose. This was not a random sample as the equipment was available at only one study site (Figure 3).

### Laboratory Procedures

Blood smears (thin and thick films) were prepared using Giemsa staining and 200 fields were read before being declared negative. Complete blood counts were determined using a quality checked Sysmex pocH-100i automated haematology analyzer and haematocrit read on a capillary tube sample centrifuged at 1,500 rpm for 3 min. Blood spots on Whatman 3M filter paper were prepared for PCR genotyping by assessment of allelic variation at three loci (merozoite surface proteins 1 and 2 and glutamate-rich protein) to distinguish recrudescence from reinfection [28].

At delivery, malaria blood smears and PCR spots (from the same sample) were prepared from the mother's peripheral blood, cord blood, placenta blood, and the baby's heel prick (Figure 2). The blood sampling of the placenta involved making a 1 cm incision half-way between the site of cord insertion and edge of the placental disc on the maternal surface. The blood that pooled into this incision was used for malaria smear and PCR genotyping.

### Pharmacokinetic Sampling

Initially a nested substudy in 13 patients was conducted to characterise the pharmacokinetic properties of both artemether (and dihydroartemisinin) and lumefantrine (Figure 3). The results of the this pharmacokinetic evaluation have been reported [23]. The efficacy, safety, tolerability, and pregnancy outcomes of AL in these women are included in this article. This was followed by a nested population pharmacokinetic study of lumefantrine (to be reported elsewhere) in 103 patients and collection of day 7 capillary plasma lumefantrine samples in 85 patients (reported here) (Figure 3). Samples were taken from a finger prick sample into four haematocrit tubes. Plasma was separated and stored at −30 °C until lumefantrine concentrations were analyzed using a validated HPLC method described previously [29].

### Objectives

The primary aim of this study was to assess the efficacy of AL in the treatment of uncomplicated falciparum malaria in the second and third trimesters of pregnancy compared to AS7 monotherapy, the standard first-line treatment at the time. The tolerability and safety of AL were also assessed as compared to AS7. The AL efficacy data were then related to day 7 plasma concentrations of lumefantrine, known to be a major determinant of parasitological response [30].

### Outcomes

#### Parasitological cure.

The primary endpoint was efficacy defined as the P. falciparum PCR-adjusted cure rates assessed at delivery or by day 42 if this occurred later than delivery, as estimated by Kaplan-Meier survival analysis. The intention-to-treat (ITT) population was defined as all randomized patients who took at least one dose of study treatment. The per-protocol (PP) population was defined as all patients completing the expected number of days of treatment who fufil the inclusion/exclusion criteria of the protocol and who were not absent for more than two consecutive weeks of follow-up by day 42 or more than three consecutive weeks of follow-up by delivery. Patients who were lost during follow-up were censored at the last day seen. Patients who had a PCR-confirmed novel infection were censored on the day of reinfection and classified as a treatment success up to that day. Patients who had a PCR-confirmed recrudescent infection were censored on the day of parasite reappearance and classified as a treatment failure. Both ITT and PP analyses make the assumption that patients who were absent from the weekly visits were malaria smear negative. Patients who developed P. vivax infection and were therefore treated with chloroquine, and patients treated with antibiotics with antimalarial activity during follow-up were not censored in the primary or day 42 study endpoint analysis.

Secondary endpoints for the mother were P. falciparum PCR-adjusted cure rates assessed at day 42; cure rates for reappearance of any malaria species (P. falciparum or P. vivax) by delivery or day 42 if later; time to fever and parasite clearance; mother peripheral blood, placenta blood, and cord blood malaria parasite positivity rates at delivery; and the day 7 capillary plasma lumefantrine concentrations in the AL group. Safety and tolerability endpoints included the incidence of anaemia, ECG abnormalities, haematological and biochemical disturbances, rates of vomiting and other AEs, pregnancy outcomes, and the physical and developmental assessment of neonates at birth and infants followed up to 1 y of age. The appearance of symptoms post-treatment was examined after excluding patients who had the symptoms on enrolment.

#### Sample size calculation.

Based on previous drug efficacy studies in this area [11,18] AL was expected to be superior to AS7. A sample size of 125 women in each group allowed detection of a difference in cure rates of 16% and 4% to be detected with 95% confidence and 80% power, allowing a 10% drop-out rate.

#### Randomisation.

Patients were assigned to one of the two treatment groups in blocks of ten using a list of random numbers. The randomization list was generated by an independent statistician who was not involved in conducting the study.

The treatment allocation was concealed in sealed envelopes labelled with the study code by another person not involved in conducting the study.

The study envelopes were sorted by code and kept at the field sites. Once a midwife identified a patient who met the inclusion criteria she was assigned the next available code. The envelope was then opened and the patient treated according to the treatment allocation.

#### Blinding.

Although initial treatment allocation was blinded, administration of subsequent medication was not, i.e., open-label. Laboratory staff reading the malaria smears had no knowledge of the treatment received. All additional sample investigation such as PCR genotyping, and patient investigation such as neurological testing, was performed by individuals who were blinded to the treatment allocation.

### Statistical Methods

Normally distributed continuous variables were compared using analysis of variance, and non-normally distributed continuous variables were compared by means of the Mann-Whitney U test. Differences in proportions were compared using the Chi-squared test or Fisher's exact test when appropriate. Statistical programs used were SPSS for Windows, version 11.0 (SPSS), and Epi Info, version 3.4.0 (US Centers for Disease Control and Prevention).

Kaplan-Meier survival analysis was used for estimation of PCR-adjusted cure rates and the log-rank test for comparison of the primary study endpoint. The follow-up time was calculated in days from enrolment to censoring, i.e., delivery or day 42 (if later), parasite recurrence or loss to follow-up.

As some of the recruited patients had a previous malaria infection documented during the pregnancy prior to enrolment, the primary endpoint was re-examined after women were classified according to their enrolment infection (primary infection or PCR-confirmed novel or recrudescent infection). Kaplan-Meier survival analysis was repeated as described previously (using the ITT population) with the addition of strata: enrolled with a primary infection, novel infection, or recrudescent infection.

Definitions used for analysis included the following: a case of uncomplicated falciparum malaria was defined as slide-confirmed P. falciparum malaria, with an asexual parasitaemia (between six parasites per 500 white blood cells and 40 per 1,000 red blood cells [RBCs], approximately equivalent to 96–150,000 parasites/μl), in the absence of signs of severe malaria. Hyperparasitaemia was defined by a parasitaemia of ≥ 40 parasites per 1,000 RBCs. Anaemia was defined as a haematocrit of less than 30%, and severe anaemia by a haematocrit below 20%. Gametocyte carriage was measured as the number of weeks during which gametocytes were seen in the peripheral blood, divided by the total number of follow-up weeks, and expressed per 1,000 person-weeks (person–gametocyte-weeks), in patients who did not have gametocytes on enrolment.

## Results

Between 23 April 2004 and 29 August 2006, 252 women with 253 episodes of acute falciparum malaria (one woman randomised twice to AS7) were enrolled; 128 patients were randomised to receive artesunate (AS7) and 125 to receive artemether-lumefantrine (AL) (Figure 1). The average gestational age (range) of patients at enrolment was 24 (13–39) wk (Table 1). Four patients did not receive a full course of treatment: three became hyperparasitaemic in the first 6 h, requiring rescue treatment (including re-randomised woman) and one developed artesunate allergy. Only eight (three AS7 and five AL) women defaulted from the study before the delivery outcome was known. The last delivery date for the study was 21 January 2007 and the last infant was followed up on 12 February 2008.

**Table 1:**
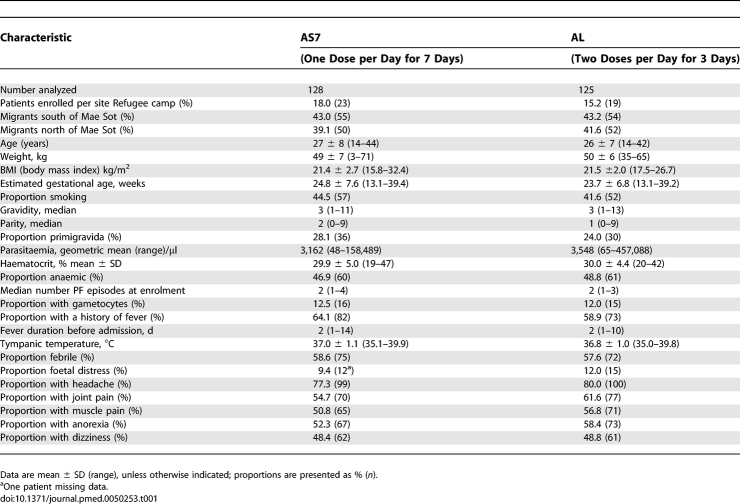
The Baseline Characteristics of Patients Analysed in the Trial

### Baseline Characteristics

There was no significant difference in the baseline characteristics of the patients in the two groups (Table 1). The median (range) total artemether and lumefantrine dose received per treatment course was 9.8 (1.5–13.7) mg/kg and 58.8 (8.7–82.3) mg/kg, respectively, and the corresponding dose of artesunate in the comparator group was 14.0 (4.0–16.0) mg/kg. More than half the patients had P. falciparum and/or P. vivax infection treated during the pregnancy before entry to the study (Table 2). There were 134 (AS7 group) and 134 (AL group) infections of P. falciparum or P. vivax documented before enrolment to the trial (Table 2). No significant differences were observed in the number of malaria infections, the total antimalarial treatments received or the PCR genotyping classification of the type of infection with which the patient entered the study (Table 2). The majority of antimalarial treatment before enrolment to the study was quinine (and chloroquine for P. vivax) but 18 of the prior treatments contained an artemisinin derivative (Table 2).

**Table 2:**
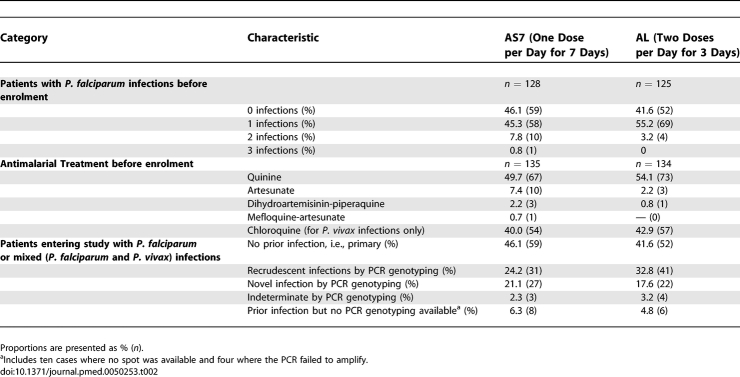
Summary of Episodes of Malaria in Patients before Enrolment

More patients in the AS7 group (22.7% [27/119]) than in the AL (11.1% [13/117]) group delivered before day 42 of the follow-up, *p* = 0.018; however, the median (range) days of follow-up until they were censored in the AS7 and AL groups, both by delivery (or day 42 if later) by day 42, were not significantly different: 60 (7–193) d and 70 (3–188) d, *p* = 0.79; and 42 (7–44) d and 42 (3–43) d, *p* = 0.11, respectively.

### Fever and Parasite Clearance

Patients in the AS7 and AL groups responded rapidly to treatment and had similar median (range) fever and parasite clearance times: 1 (1–2) d versus 2 (1–3) d, *p* = 0.20 and 2 (1–5) d, for both groups, *p* = 0.95, respectively.

### Efficacy Endpoints

**Figure 4:**
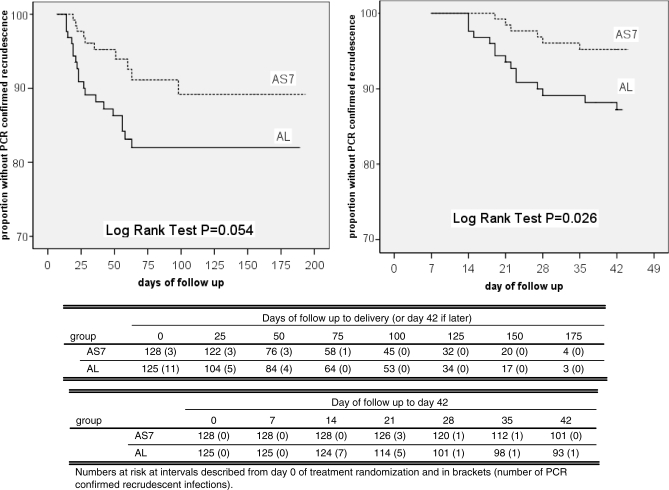
P. falciparum PCR-Adjusted Cure Rates at Delivery (day 42 if Later) (Left Graph) and at Day 42 of Follow-up (Right Graph) as Estimated by Kaplan-Meier Survival Analysis

Before delivery (or day 42 if later), there were 74 episodes of first reappearance of P. falciparum, of which 30 (41%) (AS7 = 10, AL = 20) were recrudescent infections, 44 (59%) (AS7 = 21, AL = 23) were novel infections (Table 3) and none was reported as indeterminate. The results obtained for the ITT population for PCR-adjusted cure rates have been detailed for the primary (delivery or day 42 if later) and secondary efficacy endpoints (day 42), and indicate that that AS7 was more efficacious than AL (Figure 4; Table 4). The PP analysis produced very similar results (Table 4).

**Table 3:**
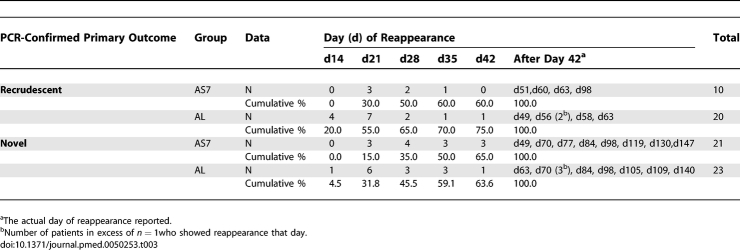
Summary of the Days of Reappearance (Recrudescent and Novel) According to the Treatment Groups until Delivery or Day 42 if Later

**Table 4:**
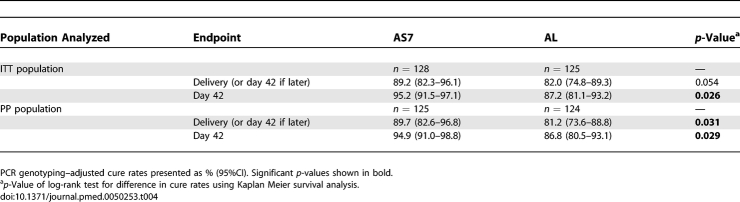
*P. falciparum* PCR-Adjusted Cure Rates (%) for the Primary (Delivery or Day 42 if Later) and Secondary (Day 42) Study Endpoints by Kaplan-Meier Analysis for the ITT and PP Populations

For the PP analysis the number of patients censored for weeks of sequential absence was similar in the AS7 and AL groups: six in each group by day 42, and eight and 12 in each group by delivery (or day 42 if later), *p* = 1.000 for both comparisons. The censored patients included two with late parasite reappearance: one (AS7 group) PCR-confirmed recrudescent infection on day 51 and one (AL group) PCR-confirmed new infection on day 140. PP PCR-adjusted cure rates by delivery (or day 42 if later) were again higher for AS7 than AL (Table 4) and likewise for PCR-adjusted cure rates by day 42 (Table 4).

The median (range) interval to recrudescent infection, 27 (14–98) d, was shorter than the time to reinfection, 37 (15–147) d, *p* = 0.015. There was no significant difference in time to recrudescence between the AS7 and AL groups 32 (19–98) d and 23 (14–63) d respectively, *p* = 0.14 (Table 3). Nearly one-third (30% [9/30]) of recrudescent infections occurred after day 42 (Table 3).

#### Cure rates based on classification of enrolment infection.

**Figure 5:**
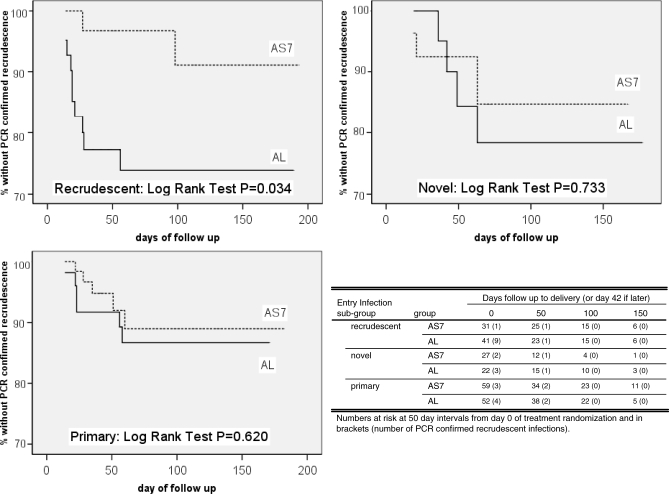
P. falciparum PCR-Adjusted Cure Rates (ITT) Assessed at Delivery (Day 42 if Later) as Estimated by Kaplan-Meier Survival Analysis after Classification of the Enrolment Infection Enrolment infections were classified by PCR genotyping into recrudescent, novel, or primary infections.

Less than half 43.9% (111/253) of the patients who entered the trial had a primary infection. The remaining patients (*n* = 142) had recurrent infections at enrolment: of whom most had one previous infection (*n* = 127), 14 had two, and one had three previous episodes of P. falciparum within the same pregnancy but before entry to the study (Table 2). PCR genotyping of blood spots from previous episodes before study enrolment permitted sub-classification of these trial entry infections into confirmed recrudescent 28.5% (72/253), new infections 19.4% (49/253) or unknown 8.0% (21/253); 11 AS7 and ten AL) (PCR indeterminate *n* = 7, or PCR spot unavailability or failure to amplify *n* = 14). Patients with unknown results were omitted from this subgroup analysis. The PCR-adjusted cure rate for the patients who entered the study with a recrudescent infection was significantly higher for AS7 compared to AL (Figure 5; Table 5). By contrast, the PCR-adjusted cure rates of AS7 and AL in patients who entered the study with new (Figure 5; Table 5) or primary infections (Figure 5; Table 5) were very similar. Thus the difference in cure rates in the overall analysis appears to be explained by the inferior cure rates of AL in treating recrudescent infections.

**Table 5:**
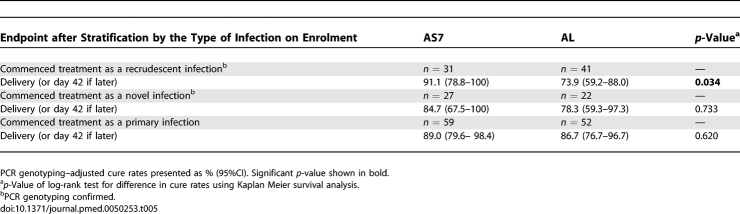
*P. falciparum* PCR-Adjusted Cure Rates (%) in the ITT Population for the Primary (Delivery or Day 42 if Later) and Secondary (Day 42) Study Endpoints after Stratification by the Type of Infection (also PCR Confirmed) on Enrolment

### P. vivax Appearance

P. vivax occurred in 36.7% (47/128) and 29.6% (37/125) of patients treated with AS7 and AL, respectively (*p* = 0.23), at a median (range) of 29 (14–125) d and 34 (14–105) d following treatment (*p* = 0.47). After patients with P. vivax infection during follow up were censored, the PCR-adjusted cure rates for P. falciparum infection at delivery (or day 42 if later) using survival analysis were improved slightly: AS7 92.2% (95% confidence interval [CI] 86.1%–98.3%) versus AL 83.8% (95% CI 76.1%–91.5%), *p* = 0.045 (Table 4).

**Figure 6:**
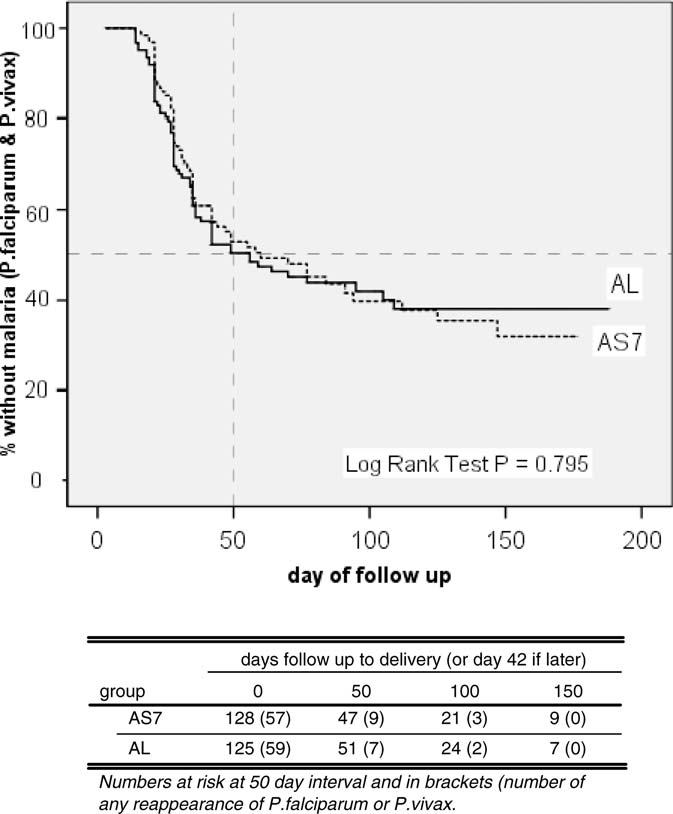
Malaria (P. falciparum or P. vivax) Cure Rates Assessed at Delivery or Day 42 (if Later) as Estimated by Kaplan-Meier Survival Analysis

A survival analysis in which any subsequent malaria infection (P. falciparum or P. vivax) appeared during follow-up to delivery (or day 42 if later) was considered a “failure” resulted in low infection-free rates of AS7 31.9% (95% CI 20.1%–43.7%) and AL 38.1% (95% CI 28.1%–48.1%), *p* = 0.795, which illustrates the limited post-treatment prophylactic effects of the two regimens. By day 50, 47.8% (121/253) of patients had a reappearance of P. falciparum or P. vivax (Figure 6).

### Use of Other Drugs with Antimalarial Activity

There were 11 patients treated with drugs with antimalarial activity (azithromycin, ciprofloxacin, erythromycin, or rifampicin); in four of these cases the treatment occurred after the day of censoring for the primary study endpoint. There was no significant difference (*p* = 0.975) between the AS7 (3.1% [4/128]) and AL (2.4% [3/125]) groups in the proportion of patients treated with other antimalarials before censoring. The trends in cure rates using survival analysis were unchanged when these patients were censored on the day of receiving these drugs (unpublished data).

### Anaemia

**Figure 7:**
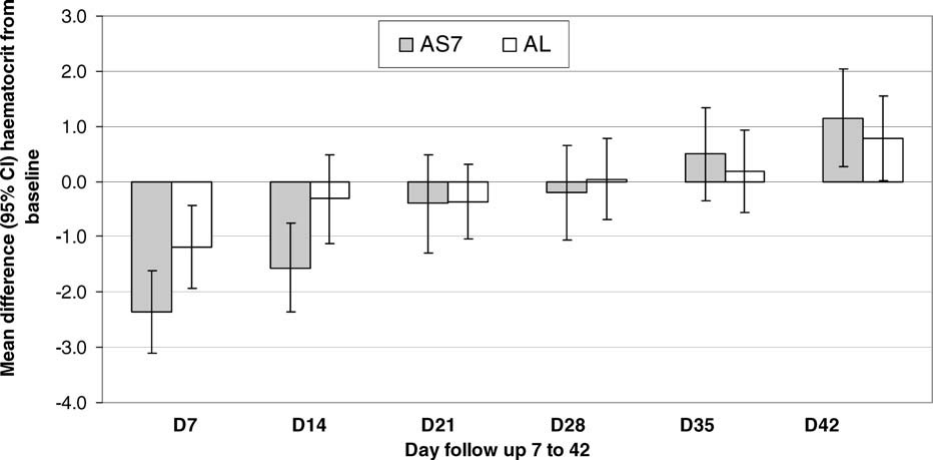
The Absolute Change in Haematocrit between Admission and Each Follow-up Day from 7 to 42 Change was calculated for each individual patient and summed as mean and 95% CI for the two treatment groups.

Nearly half the patients were anaemic at enrolment, with similar proportions in the two groups (Table 1). The absolute changes in haematocrit between enrolment and each of the follow-up days (7–42) were calculated for each patient and were summarised as mean (95% CI) values for each treatment group (Figure 7). The drop in haematocrit from enrolment was significantly greater in the AS7 group at day 7 (*p* = 0.037), but there was no significant difference by day 14 (*p* = 0.058) through to day 42 (*p* = 0.621). Nine patients (3.6%) were transfused (six AS7 group and three AL group) for malaria-related anaemia.

### Gametocyte Carriage

There was no significant difference in the proportion of patients with gametocytaemia on admission (Table 1). There were five (AS7) and one (AL) patients who developed gametocytaemia during follow-up (day 7 to day 42) with carriage of 10 (95% CI 4–21) and 2 (95% CI 0–10) person–gametocyte-weeks per 1,000 person-weeks respectively, *p* = 0.13.

### Day 7 Capillary Plasma Lumefantrine Levels

**Figure 8:**
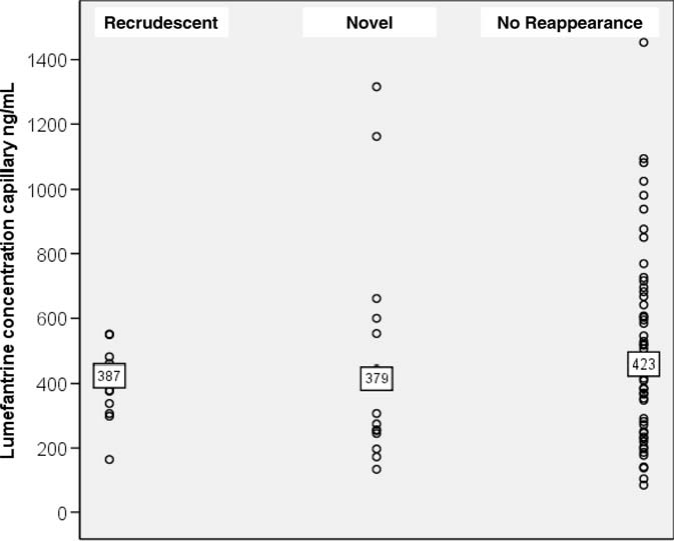
Scatterplot of Lumefantrine Concentration from Capillary Sampling on Day 7 Data are grouped according to the endpoint (recrudescent, novel infection or primary infection) after AL treatment. Geometric mean (ng/ml) displayed within text box.

In total 85 patients in the AL group had capillary plasma lumefantrine concentrations measured on day 7 following the start of treatment. The geometric mean (range) lumefantrine concentrations in patients who had subsequent recrudescent P. falciparum infections (*n* = 14) was 387 (164–551) ng/ml and 379 (134–1,320) ng/ml in those with recurrent novel infections (*n* = 17), and 423 (84.4–1,450) ng/ml in patients who had no parasite reappearance (*n* = 54), *p* = 0.26 (Figure 8).

The lumefantrine concentration (LC) is known to be higher in capillary plasma (CP) than venous plasma (VP). This relationship has been described previously by the following equation derived from data obtained at this study site [31]:


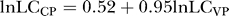


In total 35% (30/85) of patients had day 7 capillary plasma concentrations < 355 ng/ml (i.e., corresponding to 280 ng/ml in venous plasma), a threshold previously associated at this site with increased treatment failure rates in non-pregnant patients [30].

**Figure 9:**
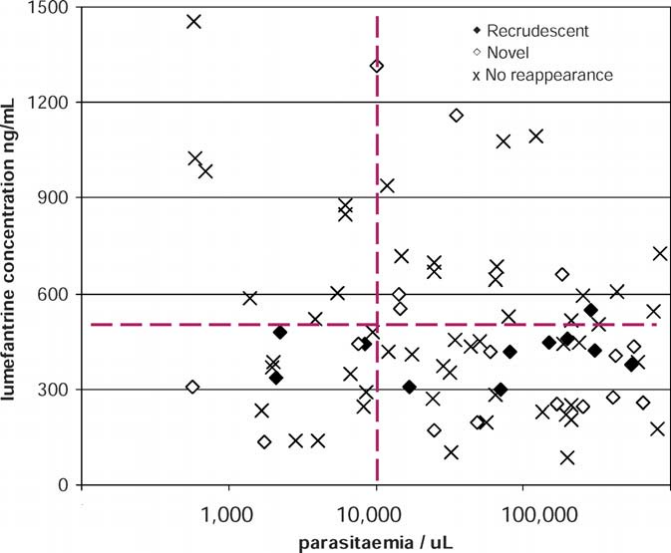
Scatterplot of Day 7 Lumefantrine Concentrations Versus Enrolment Parasitaemia Units are ng/ml. Data on *y*-axis are from capillary sampling; *x*-axis is the enrolment parasitaemia (per μl). Markers identify the primary efficacy endpoint of AL treatment: recrudescent (solid diamonds), novel infections (open diamonds) and no reappearance (×).

The majority of patients with recrudescent infections had admission parasitaemias over 5,000/μl and day 7 capillary plasma lumefantrine concentrations below 500 ng/ml (Figure 9). This is reflected in the concentration of black diamonds representing recrudescent infections in the lower right quadrant of the scatter plot (Figure 9). All 21 (100%) patients with levels over 600 ng/ml were cured, whereas 14 of 64 (22%) patients with lower values had recrudescent infections (*p* < 0.001).

### Serious Adverse Events

There were 142 serious AEs (SAEs) including one maternal death due to haemorrhagic shock from a ruptured uterus. This was considered unrelated to the AL treatment she had received 5 mo earlier. The only SAE that was definitely drug related was a markedly raised parasite count on the second day of AL treatment (parasitaemia day 0: 1.2% infected red blood cell [IRBC] to day 1: 5.4% IRBC). This patient was monitored with blood smears every 6 h for 24 h and, as the parasitaemia then reduced, she did not require rescue treatment, although she went on to have a PCR-confirmed recrudescence on day 22. For the remaining 140 SAEs (64 AS7 and 76 AL), the relationship to the treatment drug was rated as “possible” to “not related”. The most common SAE in this study was hospitalization (as required by protocol) to supervise the treatment of a reappearance of P. falciparum infection (*n* = 101 episodes) which was more common in the AL (*n* = 59) than AS (*n* = 42) group. Nine patients were transfused for malaria-related anaemia (three AL and six AS7), 12 patients were admitted for febrile illness (four pyelonephritis, two each group), one P. vivax + urinary tract infection (AS7 group), two acute respiratory infections (one each group), two scrub typhus (AL group), two pulmonary tuberculosis (one each group), one symptomatic P. vivax (AL group), and 18 patients had obstetric problems (five threatened pre-term labour [three AL and two AS7], three cesarean sections [all AL], five vacuum extractions and two forceps deliveries [all AS7], one mid-trimester abortion [AS7], and two stillbirths [one each group]).

### Other Adverse Events

One patient experienced a mild allergy (urticarial rash) after the second dose of artesunate which responded to oral chlorpheniramine and stopping artesunate. Early vomiting occurred in 1.2% (3/253) of patients, two AL and one AS7, all with the first dose and for each the repeat dose was tolerated.

The patients who had blood counts and biochemistry evaluated had a history of less fever and less symptoms on admission, than the patients who were not studied (*p* < 0.05). There was no significant difference between the groups' haematological and biochemical parameters at baseline. In the individual paired analysis there was no significant difference in the proportion of patients in each group who developed abnormal values on day 14 when they were not present on enrolment.

There was no difference in the baseline characteristics of patients who volunteered for ECG examination and those who did not have these performed. There were 51 patients treated with AL who had paired ECG examination (prior to treatment and 1 h after the last dose) with no clinically relevant abnormalities.

The only symptom that differed significantly between the groups in follow-up, when it was not present on enrolment, was tinnitus, which was more frequent in the AS7 group 8.5% (7/82) compared to 0% (0/86) in AL group, *p* = 0.006 (Fisher's exact). Tinnitus in these seven patients was short-lived (< 1 wk) and resolved in all cases.

### Birth Outcome

Data on birth outcome were documented for 96.8% (244/252) of women and not available for 3.2% (8/252) who were lost to follow-up before the end of pregnancy. Amongst the women with a known outcome there were 99.6% (243/244) births and 0.4% (1/244) abortions. There were 239 singleton births including two stillbirths and four twin births (all of whom were normal, alive, term infants of low birth weight in 87.5% [7/8] of cases).

There were no significant differences in the major birth indicators between the groups including birth weight, gestation, congenital abnormalities, and the newborn neurological optimality score (Table 6). The abortion occurred at 16.6 wk gestation in a 35-y-old, smoker, gravida 8, parity 5, in the AS7 group and was considered unlikely to be drug related as foetal distress was present before the drug was given. Both stillbirths were externally normal. One (AS7 group) was associated with histopathologically confirmed chorioamniotic inflammation due to recurrent, culture-confirmed Escherichia coli urinary tract infection, and the other (AL group) possibly related to consanguinity (marriage to a second cousin).

**Table 6:**
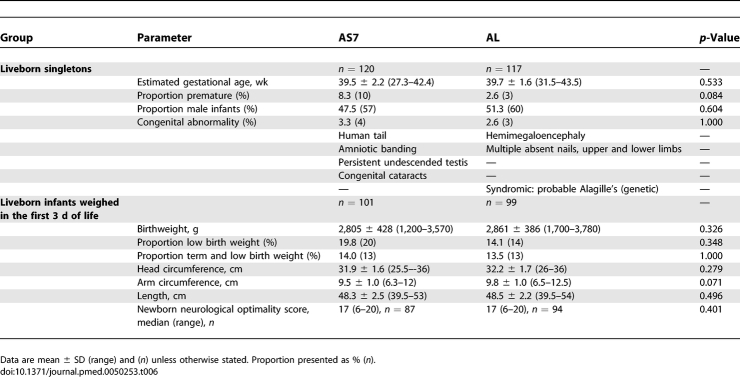
Birth Outcomes in Each Group

### Placental Malaria Infection

Malaria smear and blood spots for PCR genotyping on maternal peripheral blood, cord blood, placental blood, and the baby's peripheral blood were reported for 70% (169/243) of birth outcomes (including twins and excluding the abortion case). Concordance between microscopy and PCR detection of P. falciparum in peripheral and placental specimens was very high (Table 7). All the women with positive placenta samples had at least one reappearance of P. falciparum during follow-up after study enrolment. The single discrepant result between mother peripheral and placental blood occurred in a woman who delivered on day 7 of retreatment with artesunate and clindamycin: peripheral parasitaemia had cleared but placental parasitaemia was still detectable by PCR and microscopy. In contrast, PCR detected three positive cord blood samples when microscopy was negative. Positive cord blood samples all occurred in women with active infection at delivery (placental and peripheral smear positive), one on day 2 of treatment. All infants were peripheral blood negative (microscopy and PCR) at birth. Two of the cord blood–positive infants remained negative by weekly peripheral smear by day 28. The remaining infant, who became ill on day 20 of life, was P. falciparum positive by microscopy and PCR. Parasite genotyping of the infant blood matched the mother, cord, and placental DNA of the infection at the time of delivery.

**Table 7:**
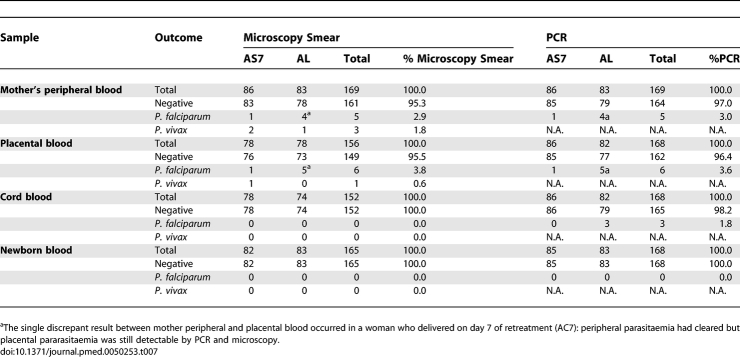
Summary of Microscopy and PCR on Delivery Samples from the Mother, Placenta, Cord, and Newborn

Three women had peripheral blood P. vivax parasitaemia at delivery, and one of these had P. vivax positive placental tissue by microscopy. No cord or baby malaria smears were positive for P. vivax by microscopy.

### Infant Follow-up to 1 Year of Life

All four sets of twins (eight babies) survived to 1 y and were developing normally. Of the singleton infants, 82.2% (195/237) were followed up for ≥ 9 mo. The main reason for loss to follow-up was leaving the study area. Overall 77.5% (93/120) of AS7 and 72.6% (85/117) of AL infants were evaluated at 12 mo, *p* = 0.32. There was no significant difference in the median months of follow-up (unpublished data). A total of nine infants died among the 237 infants followed to 1 y, an overall infant mortality rate of 38 per 1,000 live-born singletons. Significantly more infant deaths occurred in the AS7, 6.7% (8/120) than AL 0.9% (1/117) group, *p* = 0.036. Five infant deaths occurred in the first month of life (day 0–32), three of which were related to prematurity (27.3, 29.4, and 34.4 wk), while the other two were from pneumonia in the first 2 wk of life in term infants. Of the remaining infants one was premature (33.1 wk) and died of sepsis at 3 mo. The mother was diagnosed with TB and HIV postpartum. One child died from diarrhoea at 4 mo, one from suspected intraventricular haemorrhage on a background of Allagile's syndrome at 7.5 mo (AL group), and one from suspected beri-beri at 10.5 mo. There was no difference between the infants in the groups in growth parameters or developmental parameters assessed by the Shoklo Developmental Scores at 3, 6, 9 (unpublished data), and 12 mo (Table 8).

**Table 8:**
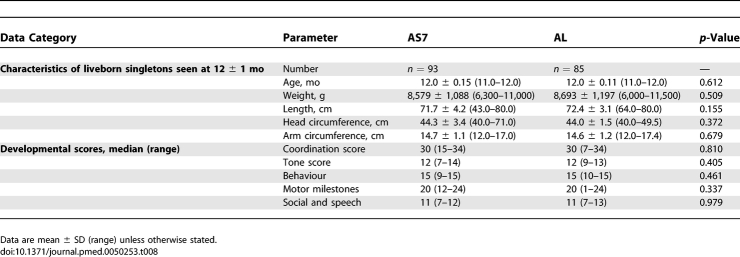
Outcome of Infants Followed up to the End of the First Year of Life in Each Group

Of 668 morbid episodes treated in 196 infants in the first year of life, acute respiratory infection 51.9% (347), skin infection 18.3% (122), and malaria 13.0% (87 including one twin) were the most common diagnoses. Malaria affected approximately one-fifth of infants in the first year of life: AS7 16.1% (19/118) and AL 19.0% (22/116), *p* = 0.61. The median (range) age of first P. falciparum was 29.9 (2.9–51) wk and 30.1 (3.4–44.3) wk for the first P. vivax infection. There was also no significant difference in the species, the median days to the first episode, or the total number of episodes in the first year of life (unpublished data), according to the mother's treatment group. The relative risk for recurrent malaria in the first year of life was significantly higher with P. vivax than P. falciparum: 2.9 (95% CI 1.2–7.0); 67.7% (21/31) versus 23.5% (4/17), *p* = 0.008.

## Discussion

ACTs are now recommended as first-line treatment for falciparum malaria throughout the malaria-endemic world, and are now specifically recommended for the treatment of falciparum malaria in the second and third trimesters of pregnancy. This is a pragmatic compromise, as ACTs may become the only available effective antimalarials, pregnant women are at increased risk from falciparum malaria, and the evidence to date indicates that artemisinin derivatives are safe in later pregnancy. This is, to our knowledge, the first randomised controlled trial of fixed-dose ACT in the treatment of uncomplicated malaria in pregnancy. Artemether with lumefantrine was very well tolerated with no adverse effects either for the mother or for the foetus, yet its efficacy was lower than expected. Both artesunate monotherapy for 7 d and the standard 3 d regimen of artemether and lumefantrine resulted in cure rates below the 90% efficacy threshold recommended by WHO [9]. Cure rates are likely to be even lower outside the setting of a trial when all doses cannot be directly supervised. Failure rates were over twice as high as those in non-pregnant adults, given the same dose in this same location, for whom AL cure rates consistently exceed 95% [11]. The cure rates in pregnant women are lower than observed in young children in this area, a patient group with little or no immunity, which suggests that the poor therapeutic response is not explained by the malaria-specific immunosuppression of pregnancy. The significantly lower cure rates with AL were largely explained by the difference in cure rates for recrudescent infections (i.e., women presenting with a recrudescence of a previous infection). This study initially recruited women with a recurrent P. falciparum infection only, as there had been no previous experience to establish the safety of AL in pregnancy. This trial was not originally designed to compare cure rates in recrudescent with those in new or primary infections (Figure 5; Table 5), but the exploratory analysis shows a clear superiority of AS7 over AL in this particular subgroup. There was no difference in efficacy between AL and AS7 in the treatment of primary or new infections. This difference between primary and recrudescent infections presumably reflects reduced class susceptibility of the recrudescent infections to quinine, mefloquine, and lumefantrine [32]. Although AL is not routinely used in the study area, there is cross-resistance with mefloquine, which is combined with artesunate in the first-line treatment in the non-pregnant population. Resistance is associated with an increase in *Pfmdr1* copy numbers [33–35]. Parasites with increased *Pfmdr1* copy number are also slightly less sensitive to artemisinin derivatives, although the differences are much less than for lumefantrine [36].

The poor cure rate of AL in pregnant compared to non-pregnant patients probably results from the lower drug concentrations of both component drugs. The pharmacokinetic properties of both artemether and lumefantrine in later pregnancy are altered substantially. As with several other antimalarial drugs, plasma concentrations of artemether, its principal metabolite dihydroartemisinin, and lumefantrine are reduced in pregnant women [23,37–39]. This was shown in the conventional pharmacokinetic study [23] embedded into the early phase of this trial, and is reflected again by the relatively low day 7 capillary plasma lumefantrine concentrations.

We have shown previously that low plasma lumefantrine levels, reflected by the area under the plasma concentration time curve (or its surrogate, the day 7 level), and parasitaemia are the two main determinants of the therapeutic response in studies conducted in non-pregnant patients [30]. Thus patients with lower drug levels and higher parasitaemias are most likely to fail treatment (Figure 9). Where drug resistant parasites are prevalent, the combined effects of low antimalarial drug levels and the attenuated antiparasitic immunity in pregnancy in patients with heavier parasite burdens (itself a reflection of reduced immunity against the infecting parasite population) increase the risk of treatment failure.

The need in malaria-endemic areas for a safe and effective drug with an adequate post-treatment prophylactic effect in pregnancy is clearly demonstrated by the fact that half of the patients in this study had recurrent malaria infection within 50 d. All women who had positive placental blood at delivery had parasite reappearance after randomization.

Several factors contribute to the poorer treatment responses during pregnancy; P. falciparum parasites sequester in the placenta and so the parasite burden is underestimated, antiparasitic immunity is compromised, and antimalarial drug levels are generally lower. This study shows that guidelines for defining antimalarial efficacy [40,41] in non-pregnant patients may not be applicable to pregnancy. Nearly one-third of PCR genotyping–confirmed recrudescent infections occurred after day 42, whereas in non-pregnant patients treated with these drugs nearly all recrudescences occur within 42 d. Importantly this happened equally with artesunate and artemether-lumefantrine. One woman harboured a single infection at sub-patent densities for 3 mo following artesunate. Thus pregnant women can harbour recrudescent infections for protracted periods. This has been reported previously in Asia [6,28] and more recently in Africa [42]. The duration of follow-up in all antimalarial drug trials in pregnant women should be extended at least to delivery, or day 42 if this comes later.

In contrast to high-transmission settings, microscopy of the placental smear did not add to the diagnostic yield. P. falciparum PCR genotyping of maternal peripheral and placental blood at delivery did not detect any submicroscopic infections. All cases of placentas that were positive by PCR were found in mothers with a positive peripheral blood smear or very recently treated positive peripheral blood smear. Early detection and treatment of malaria by active weekly screening of maternal peripheral blood smears appears to be a very reliable method to detect P. falciparum malaria in pregnancy in this area of low transmission.

There were few adverse events in this study and these were mainly unrelated to treatment. Anaemia is a significant adverse effect of malaria in pregnancy [43]. Artemisinin and its derivatives temporarily suppress erythropoeisis, but this “trades off” against the benefit of rapidly reducing parasitaemia and thereby reducing malaria-associated haemolysis. The slightly lower levels of haematocrit and haemoglobin at day 7 in the AS7 group were expected because of the longer haematopoietic suppression from the 7 d regimen [44]. This was not clinically significant as there was no difference in day 42 haematocrit levels, or final haematocrit before delivery.

No pregnant women or newborns in this study died from malaria. The newborn anthropometric results and neurological scores (Table 5) were very similar in the two groups and are consistent with having multiple malaria infections in pregnancy [18,43,45,46]. The better efficacy of AS7 compared to AL did not result in improved birth outcomes in the AS7 group nor would one expect it to, as this study was not powered to detect such differences. This was a study conducted in the second and third trimesters so any congenital abnormalities are most unlikely to be drug related and the rates were similar to previously reported studies in this area [18,45]. Although there were significantly more infant deaths in the AS7 group, these deaths were unrelated to treatment. The growth parameters and Shoklo developmental score of the infants during and up to 1 y of follow-up were also reassuring. Overall the 252 women in this study were treated with a total of 362 artemisinin-based treatments for slide-confirmed P. falciparum infections before and during this study. This provides further reassuring safety data on the use of artemisinins in the second and third trimesters of pregnancy; however, further large-scale assessments are needed to confirm this safety profile.

One of the limitations of this trial includes the lack of blinding to antimalarial treatment administration. A placebo for this trial would have been difficult as the two regimens were very different. The embedded pharmacokinetic sampling would have been impossible without unblinding the trial or sampling women from both groups to maintain blinding, which would be unethical. The ad-hoc analysis shows differences within the strata, rather than simply between groups, which suggests the lack of blinding did not influence the efficacy results.

Immunity contributes significantly to treatment outcomes. It is probable that treatment responses to AL would be better in higher-transmission settings. But in this setting where P. falciparum is relatively resistant and transmission intensity (and therefore immunity) is low, the cure rates with AL in pregnancy are unsatisfactory. A dosage increase is needed. A major drawback is that lumefantrine absorption has been shown to be dose-limited [47], so simply increasing the individual doses is unlikely to result in higher cure rates. A longer or more frequent AL treatment course should now be evaluated. Importantly, the most favourable efficacy results for this drug in non-pregnant patients on the Thai-Burmese border were obtained by giving six doses over 5 d [48]. A 5 d regimen would provide artemether exposure over three parasite asexual cycles and 67% more lumefantrine. Pharmacokinetic dose evaluation studies on higher AL dose regimens should now be performed. Adequate cure rates for falciparum malaria in pregnancy will only be achieved when the doses are optimised specifically for pregnancy. Antimalarial efficacy studies in pregnancy are more complex than in non-pregnant patients. Lengthy follow-up is needed both to capture delayed recrudescence and for the assessment of the newborn. A safe and effective, short course, artemisinin-based combination treatment for uncomplicated P. falciparum malaria in pregnant women has yet to be found.

### Generalisability

Our results do not indicate that the conventional regimen of AL cannot be used to treat pregnant women with uncomplicated falciparum malaria in other endemic regions. However, they show that in an area of multidrug resistance, the lower lumefantrine plasma levels found in pregnant women will result in a lower parasitological efficacy. In areas of higher endemicity where multigravid pregnant women have some protection from acquired premunition, the conventional regimen of AL, i.e., two doses per day for 3 d, may be more effective, and studies (including pharmacokinetics) are in progress at the time of writing.

### Overall Evidence

There are no data on a direct comparison of the pharmacokinetics of lumefantrine in pregnant and non-pregnant women. The only comparable pharmacokinetic data from the same population and the same pharmacology laboratory are from adults (mostly males), and these were used in the present discussion. The results of the population kinetic study of lumefantrine in the pregnant women of this study will be presented elsewhere. Preliminary analysis confirms the findings of the initial pharmacokinetic study [23] and show that plasma levels of lumefantrine are generally lower in pregnant women.

### Conclusions

The current standard six-dose artemether-lumefantrine regimen was well tolerated and safe in pregnant Karen women with uncomplicated falciparum malaria, but efficacy was inferior to 7-d artesunate monotherapy and was unsatisfactory for general deployment in this area. Reduced efficacy probably results from lowered drug concentrations in later pregnancy in the presence of relatively resistant parasites. A higher-dose regimen may be needed to treat pregnant women effectively and should now be evaluated. Longer follow-up is needed when assessing antimalarial drugs in pregnancy as recrudescences may occur months after administration of rapidly eliminated drugs.

## Supporting Information

Text S1 CONSORT Checklist (115 KB PDF)

Text S2 Trial Protocol Artemether-lumefantrine treatment of multidrug-resistant falciparum malaria in pregnancy: a pilot study.
(198 KB DOC)

## Abbreviations

ACT: artemisinin combination therapy
AE: adverse event
AL: artemether-lumefantrine 3 d
ANC: antenatal clinic
AS7: artesunate 7 d
CI: confidence interval
ECG: electrocardiograph
IRBC: infected red blood cell
ITT: intention to treat
PP: per protocol
RBC: red blood cell
SAE: serious adverse event
SMRU: Shoklo Malaria Research Unit
