# Supplementary material for: A Randomised Controlled Trial of Artemether-Lumefantrine Versus Artesunate for Uncomplicated Plasmodium falciparum Treatment in Pregnancy
Source: PLoS Med. 2008 Dec 23;5(12):e253. doi: 10.1371/journal.pmed.0050253 (PMC2605900; doi:10.1371/journal.pmed.0050253)
Supplement: Text S2 — Artemether-lumefantrine treatment of multidrug-resistant falciparum malaria in pregnancy: a pilot study. (198 KB DOC) [file pmed.0050253.sd002.doc]

**Title:** **Artemether-lumefantrine treatment of multi-drug resistant falciparum malaria in pregnancy: a pilot study**

**Product: Artemether-lumefantrine (Coartem)**

###### Version: Revised 5 February 04

**Design: Single-blinded, controlled, randomised**

**Study site: Shoklo Malaria Research Unit, Mae Sot, Thailand**

**Principal Investigator: Rose McGready**

**Principal Co-Investigator François Nosten**

**Co investigators: Thein Cho, Napaporn Khan Keo, Katja Wustefeld, Kasia Stepnieuska, Ronatrai Ruangveerayuth, Sornchai Looareesuwan, Nicholas J White,**

**Study Directors: Melba Gomes**

**Isabela Ribeiro**

**Data handling and analysis: Shoklo Malaria Research, Mae Sot, Thailand**

**Sponsor: UNDP/World Bank/WHO Special Programme for Research and Training in Tropical Diseases (TDR)- Geneva, Switzerland**

**OXTREC application # 033-02 approved on the 07^th^ Feb 2003**

**Artemether-lumefantrine treatment of multi-drug resistant falciparum malaria in pregnancy: a pilot study**

Principal Investigators:

Rose McGready ____________________________ Date _____________

François Nosten ____________________________ Date _____________

Study Directors:

Melba Gomes ____________________________ Date _____________

Isabela Ribeiro ____________________________ Date _____________

1. **Introduction**

Multi-drug resistant falciparum malaria is a major threat to the communities living in endemic areas of SE Asia and is spreading fast to the rest of the tropical world. On the border areas of Thailand, *P. falciparum* has developed resistance to almost every antimalarial drug^1^. This poses particular problems for the treatment of pregnant women, a group especially vulnerable to *P. falciparum* infections^2^. Chloroquine, a drug generally considered safe to pregnant women, ceased to be effective in this part of the world over 30 years ago. Sulfadoxine-pyrimethamine also became ineffective in the 1980’s and susceptibility of *P. falciparum* to quinine has declined steadily. Mefloquine was deployed in Thailand in 1984 but resistance emerged six years later and the drug is now used in combination with 3 days of artesunate in non-pregnant patients with uncomplicated falciparum malaria^3^. In our extensive ante-natal clinic programme on the NW border of Thailand, mefloquine was used initially to treat pregnant women infected with *P. falciparum* resistant to quinine, but in very large and detailed studies we described an increased risk of stillbirth associated with its use in pregnancy^4^. We can therefore no longer use it. The only drug available to treat uncomplicated *P.falciparum* infections in pregnant women remains quinine (30 mg/kg/d for seven days). Quinine is unsatisfactory as first-line therapy in this area and new treatments are required^5^. It is associated with 20-30% recrudescence rates even with observed treatment, frequent side effects such as tinnitus, and more seriously hypoglycaemia, and consequently with a poor adherence (compliance) resulting in an even lower efficacy. In our experience, artesunate (12 mg/kg over 7 days) is safe and effective in the treatment of recrudescent infections following quinine^6^ and we have so far treated with artesunate and followed prospectively, over 600 pregnant women and documented the outcome of pregnancy and child development (the largest series to date). We found no evidence of toxicity to the mother or the foetus and detailed evaluations of the newborn have all been normal to date^7^. However over 17% of patients do have recrudesce after one, or even several courses of artesunate and adherence to this seven day regimen is poor when drug intake is not supervised. A better (3 days) second line treatment is clearly needed.

1. **Rationale**

Coartemether is a fixed combination of artemether and lumefantrine, the result of research undertaken by Chinese scientists, and developed into a GMP product by Novartis Pharma. It has been extensively studied and has proved remarkably safe and effective against multi-drug resistant *P.falciparum* infections. The largest GCP studies on the safety, efficacy and pharmacokinetics of Coartemether were conducted at SMRU^8-12^. However there is no experience with the combination in human pregnancy. In animal studies, lumefantrine was shown to be neither mutagenic, nor embryotoxic. Doses up to 1000 mg/kg have tested and shown to be well tolerated, with no evidence of maternal or embryo/foetal toxicity. Artemether, however, showed maternal effects and embryo-foetal toxicity at 10 mg/kg in rats but no teratogenicity, with a no adverse effect level of 3 mg/kg. Similarly, embryo-foetoxicity was documented in rabbits at 30 mg/kg, with no evidence of teratogenicity. The no adverse effect level in rabbits was 25 mg/kg suggesting a steep dose-response curve for embryotoxicity for this specie. Additional studies were conducted with the combination (ratio 1:6) of artemether and lumefantrine. In rats receiving doses of 0, 30, 100 and 300 mg/kg of artemether/lumefantrine, reduced maternal body weight and food consumption and high level embryotoxicity was seen with doses greater than 100 mg/kg. Doses of the combination of artemether/lumefantrine of 30 mg/kg were neither embryotoxic or teratogenic. In rabbit studies, a more favourable profile was seen. Doses greater than 210 mg/kg of this combination were associated with embryo-foetal lethality. Viable foetuses, however, showed no abnormalities. Doses up to 105 mg/kg were free of treatment-induced effects. In summary, artemether, like all artemisinin derivatives, can induce foetal toxicity/resorption in the early days of gestation in rodents, however this would be very difficult to document in human pregnancies. Available data from clinical studies suggests that the use of artemisinin drugs later in the pregnancy is safe^7^. This evidence was the basis for the recent recommendation that artemisinin derivatives could be used in the second and third trimesters of pregnancy if no other suitable treatment was available^13^. Coartemether tablets (20/120 mg of artemether/ lumefantrine) is effective as a 3 days regimen provided that it is co-administered with some fat^11^ to ensure adequate lumefantrine absorption. Indeed the day 7 lumefantrine plasma level is the main determinant of efficacy in non-pregnant patients. However the kinetics of lumefantrine may be altered in pregnancy. We propose to study the efficacy and the kinetics of lumefantrine in pregnant women treated with Coartemether for uncomplicated *P.falciparum* infections that failed to be cured by quinine and for whom we have no effective alternative. This is a necessary prelude to large scale clinical evaluation. WHO recognises the urgent need for improved treatment of multi-drug resistant malaria in pregnancy, and the lack of information on drugs which are effective and available for the treatment of pregnant patients. This is of particular relevance to this population, living in an area of multi-drug resistant falciparum malaria. An effective, simple and well tolerated antimalarial treatment for pregnancy is urgently needed.

1. **The SMRU ante-natal clinics (ANC)**

The Shoklo Malaria Research Unit has acquired a unique experience in studying the epidemiology, the prevention and the treatment of multi-drug resistant *P. falciparum* malaria in pregnancy. SMRU has conducted the largest treatment studies to date, using quinine, mefloquine and artesunate. This has been achieved through the ANC system put in place in 1986. The aim of the weekly ANC is to detect and treat all parasitaemic episodes during pregnancy through the weekly screening. At each visit the women are examined by trained Karen midwives and a blood smear is taken for the detection of malaria parasites. Every two weeks, an haematocrit is measured. Women routinely receive ferrous-sulphate and folic acid treatment doses from the 1^st^ ANC consultation until delivery, and those with severe, anaemia (Ht<20%) receive Hepatitis B and HIV screened blood transfusion. All women have an obstetric ultrasound at their first ANC attendance and a dating scan at 18 [16-20] weeks. The women are encouraged to come and deliver under supervision in the SMRU facilities. Complicated deliveries requiring Caesarean section are referred to Mae Sot hospital (1 hour drive). At birth the labour is monitored using a partogram and after delivery the newborn is examined for the gestational age (if no 18-week ultrasound has been done) and for neurological development using a system of tests adapted for us by Dr. L. Dubowitz. This system of ANC is attended by more than 90% of the pregnant women living in the camps. It has resulted in almost complete prevention of malaria related maternal mortality, and a significant improvement in the health of pregnant women and the newborn. All the data collected has been entered into databases and this represents the largest set of information on malaria and pregnancy with over 20,000 pregnancies documented. Three expatriate doctors and 30 local staff work in the ANC, in addition to 17 experienced Karen laboratory technicians. A part-time experienced obstetrician (KW) will complete the team of investigators and supervise the midwives. The proposed studies will be conducted within these established clinics.

1. **Objectives**

To define the efficacy, the safety and the pharmocokinetics of the combination artemether-lumefantrine (AL) in the treatment of recrudescent but uncomplicated *P. falciparum* infections in the last two trimesters of pregnancy in an area of multi-drug resistant falciparum malaria.

1. **Study methodology**
2. **Population and sample size**

Pregnant women with recrudescent *P. falciparum* infections, but with no sign of severity in the second and third trimester of gestation will be recruited provided they give written informed consent. This will be a randomised open-label comparison of 3 days artemether-lumefantrine versus the current standard recommended treatment: artesunate given for seven days. A sample size of 100 women in each group will allow to detect a 3 fold difference in cure rate, assuming 16% and 4% failure rates in the artesunate and Coartem ® group respectively^6^, with 90% confidence and 80% power and 5% drop out. A total of 250 women will allow a 20% drop out rate, the maximum observed in our experience. Twelve study patients will be asked to participate to a formal pharmacokinetic study of lumefantrine and artemether (see below). All remaining patients in the Coartem group will be asked to volunteer to a population–pk study (see below). In a sub-group of 50 women 2 additional blood specimens will be taken (day 0 and 14) for biochemistry, liver and renal function tests.

1. **Definitions**

- Clinical

An uncomplicated case of malaria will be defined by the absence of signs of severity as listed in the appendix 1. Low birthweight will be defined by a birthweight <2500 gr. A term delivery by a gestational age at birth ≥37.0 weeks, a pre-term delivery by a gestational age ≥ 28 and <37 weeks, and an abortion by a delivery < 28 weeks. Gestational age will be estimated using biparietal diameter, abdominal circumference and femur length measured by ultrasound at 18 ± 2 weeks. If ultrasound measurement is not available gestational age will be estimated by fundal height using the formula established for this population [GA (weeks)= FH (cm) X 0.997 + 4.968], and by the Dubowitz score at delivery. A symptomatic malaria case will be defined by the presence of a temperature (tympanic temp>37.5^o^C) or a history of fever in the previous 2 days with one or more of the following symptoms: headache, muscle/join pain, nausea/vomiting in a pregnant women together with asexual forms of *P. falciparum* on the microscopic examination of the peripheral blood.

- Laboratory

A case of malaria will be defined by the presence in the peripheral blood of asexual forms of *P. falciparum* (with a parasitaemia > 5 per 500 WBC to avoid difficulties in parasite identification) in a pregnant woman. Blood smear (thin and thick films) will be prepared using the Giemsa coloration and will be read for 200 fields before declared negative. Parasite count will be given by 500 wbc and by percentage of infected red cells for counts above 1000 parasites per 500 wbc. All stages of the parasites will be recorded (asexual and gametocytes). Anaemia will be defined by an haematocrit < 30% and severe anaemia by an haematocrit <20%. The gametocyte carriage will be defined as the number of weeks during which gametocytes are seen in the peripheral blood, divided by the total number of follow-up weeks and expressed per 1000 woman-weeks.

- Parasitological cure

An episode of falciparum malaria will be declared cured if the peripheral blood of the women remains free of parasites after treatment and until delivery or day 42 whichever comes last. In case of recrudescence within the follow-up period, the distinction between a re-infection and a true recrudescence will be made by parasite genotyping using the PCR technique used at this site and described previously^14^. Technicians and microscopists will be unaware of the treatment given.

1. **Inclusion/exclusion criteria**

- **Inclusion**:

Pregnant women with uncomplicated falciparum infection or mixed infection (i.e. *P. falciparum* & *P. vivax*), symptomatic or not, in the second (EGA≥13.0 weeks by ultrasound or fundal height ≥ 12 cm if ultrasound not available) or third trimester, who have failed after a course of quinine for seven days, who are willing and able to participate and comply with the study protocol, attend the SMRU ANCs regularly and agree to delivery at SMRU.

- **Exclusion**:

Splenectomy, a known chronic disease (cardiac, renal, hepatic, known haemoglobinopathy (alpha-thalassemia, β-thalassemia, G6PD deficiency), known hepatic or renal impairment, inability to follow the ANC consultation, history of alcohol or narcotic abuse, inability to tolerate oral treatment, vomiting any of the treatment doses, severe malaria (see appendix 1) and allergy to artemisinin derivatives or any of the study drug. Patients with an haematocrit < 25 % will be excluded from the formal kinetic study.

1. **Study procedures**

- **Admission:**

Pregnant women attending the SMRU ANCs, who had received previously a course of quinine for the treatment of the falciparum malaria, will be invited to participate in the study if they present again with another episode of uncomplicated *P. falciparum* malaria. Each patient will be explained the purpose of the study and if they consent, will be admitted in the study, and will be treated with artemether-lumefantrine or with artesunate. The first twelve of the women randomised to the coartem group that consent to the blood sampling schedule will be recruited in the formal pk study. The remaining will participate in a population-pk study.

At enrolment, a full medical history and examination (including obstetric evaluation) will be carried out by a physician and a midwife. Complete blood count, blood glucose, blood group and parasite count will be measured on admission. Thick and thin blood films will be stained with Giemsa and parasite density will be expressed per 1000 rbcs or per 500 wbcs as routinely done at the clinics. Parasite *in vitro* susceptibility to artesunate, quinine, lumefantrine, chloroquine will be measured using the DELI assay.

**Table i**: Schedule of Evaluation - mother

|  | D0 | D1 | D2 | D3 | D4 | D5 | D6 | D7 | D14 | D21 | D28 | D35 | D42 | (Wk) | Del* |
| --- | --- | --- | --- | --- | --- | --- | --- | --- | --- | --- | --- | --- | --- | --- | --- |
| Consent | x |  |  |  |  |  |  |  |  |  |  |  |  |  |  |
| Medical History | x |  |  |  |  |  |  |  |  |  |  |  |  |  |  |
| Physical Exam by physician | x |  |  |  |  |  |  | x | x | x | x | x | x | x |  |
| Parasite smear | x | X^1^ | X^1^ |  |  |  |  | x | x | x | x | x | x | x | x |
| - Haematocrit | x |  |  |  |  |  |  | x | x | x | x | X | x | x | x |
| - CBC | x |  |  |  |  |  |  |  | X^2^ |  |  |  |  |  |  |
| - Renal & hepatic | x |  |  |  |  |  |  |  | X^2^ |  |  |  |  |  |  |
| - Blood Group | x |  |  |  |  |  |  |  |  |  |  |  |  |  |  |
| - U/A | x |  |  |  |  |  |  |  |  |  |  |  |  |  |  |
| - PK sample | (x)^3^ | (x)^3^ | (X)^3^ | (x)^3^ | (x)^3^ | (x)^3^ | (x)^3^ | (x)^3^ | (x)^3^ |  |  |  |  |  |  |
| - PCR | X |  |  |  |  |  |  | (x)^4^ | (x)^4^ | (x)^4^ | (x)^4^ | (x)^4^ | (x)^4^ | (x)^4^ | X |
| - In vitro testing | x |  |  |  |  |  |  | (x)^4^ | (x)^4^ | (x)^4^ | (x)^4^ | (x)^4^ | (x)^4^ |  |  |
| Ultra-sound | x |  |  |  |  |  |  |  |  |  |  |  |  |  | X |
| EKG^2^ | x |  | X |  |  |  |  |  |  |  |  |  |  |  |  |
| Treatment (As) | x | x | x | x | x | x | x |  |  |  |  |  |  |  |  |
| Treatment (CoA) | x | x | x |  |  |  |  |  |  |  |  |  |  |  |  |
| Adverse Events^5^ | X | X | X |  |  |  |  | X | X | X | X | X | X | X | X |

* Refer to table of delivery schedules below

1 If positive and until 2 consecutive negative blood smears

2 To be performed in 50 randomly-selected women

3 Five samples at randomly selected intervals within the 14 days in the Coartemether group, participating to the population-pk study. The baseline (D0) sample for lumefantrine level will be taken in all patients(formal pk and population pk)

4 To be performed only in case of a positive falciparum smear of follow-up

5 Adverse events will be actively documented on the first 3 days, or until blood smear negative. Then the patients will be asked to report any symptoms occurring between day 2 and 7

**Table ii**: Schedule of Evaluation – Infant follow up

|  | At birth | M1 | M2 | M3 | M4 | M5 | M6 | M7 | M8 | M9 | M10 | M11 | M12 |
| --- | --- | --- | --- | --- | --- | --- | --- | --- | --- | --- | --- | --- | --- |
| Sex | X |  |  |  |  |  |  |  |  |  |  |  |  |
| Birth weight | X |  |  |  |  |  |  |  |  |  |  |  |  |
| Weight^2^ |  | X | X | X | X | X | X | X | X | X | X | X | X |
| Dubowitz score scale | X |  |  |  |  |  |  |  |  |  |  |  |  |
| Congenital Abnormalities | X |  |  |  |  |  |  |  |  |  |  |  |  |
| Motor & Neurological Assessment | X^ref.15^ |  |  |  |  |  | X^ref.15^ |  |  | X^ref.15^ |  |  | X^ref.17^ |
| Malaria Smear | X |  |  |  |  |  |  |  |  |  |  |  |  |
| PCR^1^ | X |  |  |  |  |  |  |  |  |  |  |  |  |

1 If mother peripheral blood smear positive for *P.falciparum* in the 2 weeks before birth.

2 Head, length and arm circum. At birth, 3,6,9 and 12 months

- **Randomisation procedures:**

Patients will be randomised 1:1 by blocks of 10. The computer generated randomisation list will be prepared at the study site and kept by the Study Director. Treatment category for each patient is to be provided in a sealed envelope, which is to be opened at the time of enrolment to the study by one or more specially assigned study nurses. Laboratory technicians and neuro-development tester will be unaware of the treatment(s) received by the women. A separate randomisation will determine the sampling schedule for the women participating to the population-pk study.

- **Informed consent:**

The purpose of the study will be explained in the patient's own language. She will also be given a written explanation that will be read to her if she is unable to read by herself. It will be made clear that refusal to participate at any stage will in no way alter the quality or availability of subsequent care provided.

- **Study drug regimen**:

Group 1: Artesunate (Guilin Factory no.1, Peoples’ Republic of China; 50 mg tablets) (2 mg/kg/d) for 7 days. This will be given with water and according to the woman’s weight (see Appendix 2)

Group 2: Artemether-Lumefantrine (Novartis, Basel, Switzerland) (20/120 mg artemether/lumefantrine) 4 tablets twice a day for 3 days with 200 ml of chocolate milk at each dose (see Appendix 2).

1. **Schedules: Blood samples, EKG, clinical follow up (Table i).**

- **Admission:** blood smear and haematocrit, PCR spot, parasite culture, lumefantrine baseline, haematology, blood glucose, blood group, hepatic and renal functions, total blood volume: 5 ml. Urine dipstick analysis (blood, protein, glucose).
- **Follow up:** Blood smear and haematocrit will be performed weekly by finger stick. If consent is obtained, five additional capillary specimens for lumefantrine levels in patients receiving this drug will be taken in all women of the coartem group (except in the 12 women in the formal pk study) at four random times between 1 and 14 days after drug administration for a population pharmacokinetic-pharmacodynamic assessment. In order to optimise profile definition, randomisation will be taken within the four time series: 0-72, 72-96, 96-144, and 144-336 hours. Samples randomised within 2 hours of each other will be re-randomised. Plasma will be separated and stored at -30C until analysis. For the 12 patients who agree to participate to the formal pharmacokinetic study, (5 ml) will be obtained before the first dose (day 0) [for parasite culture, PCR spots and hematology and biochemistry as described above], on day 2, an indwelling Teflon cannula will be inserted in a forearm vein, and will be kept patent with heparinised saline for 24 hours. 2 ml of blood will be drawn before the last dose and at the following times: H0.5, H1, H2, H4, H6, H8, H12, H24, and thereafter by venepuncture at H48, H72 (+3ml for biochemistry), H96, H120, H144 and H168. The maximum volume of blood drawn for drug analysis over a one week period will be approximately 36 ml. Blood samples will be centrifuged (2000 g, 10 min), and plasma will be stored at –20 °C (for lumefantrine assays) and in liquid nitrogen (for artemether and DHA assays) until drug analysis.
- Every woman in the Coartem ® arm of the study (but excluding the 12 patients participating to the kinetic study) will be asked to volunteer for an additional 3 cc venous blood sampling for biochemistry and haematology at day 14 of the follow up, until the target size of 50 paired samples (D0 and Day 14) has been reached.
- **In case of recrudescent parasitaemia:** 5 ml of blood for blood smear, parasite culture for in-vitro drug sensitivity, PCR, haematocrit, haematology.
- **At delivery:** maternal, cord, placenta and baby PCR, malaria smear and haematocrit will be done. Serum and RBCs will be stored for maternal, cord and placental samples and placental biopsies will be fixed in Streck fixative following SMRU standard SOPs (Appendix 6). Cord, maternal milk/blood, placental lumefantrine levels will be measured in women who deliver within 14 days after taking the last dose of artemether-lumefantrine. An ultrasound will be performed to ascertain foetal viability at the beginning of labour.

Table iii. Delivery sampling.

| Delivery samples | mother | cord | placenta | baby |
| --- | --- | --- | --- | --- |
| PCR blood (1ml) | X | X | x 3 | X |
| Serum and RBC 5cc | X | X | X |  |
| Malaria smear | X | X | X9 crushed | X |
| HCT | X | X | X | X |
| Biopsy |  |  | X |  |

- **Electrocardiograms:** 12 lead ECG will be performed in 50 randomly selected women in each group, at baseline and on day 2 (1hr after the last dose of the medication).
- **Ultrasound** will be performed to confirm the estimated gestation, foetal viability prior to treatment, placenta position, and amniotic fluid volume.
- **Clinical assessment and follow-up**: Patients will be kept in the antenatal In-Patient-Department in Mae La camp for 24 h and then seen daily until parasite negative; they will be then followed up weekly in the antenatal clinics. Daily evaluation, including clinical examination, drug administration, recording of subjective and objective side effects will be recorded in a case record form (Appendix 3). Thereafter the women will be seen weekly and adverse events obtained by questionnaire recorded for 6 weeks. All women recruited in the trial will be assessed monthly by an obstetrician who will also supervise the deliveries. In case of re-appearance of falciparum parasites during the follow-up period, the patient will receive artesunate-clindamycin for 7 days and be followed up weekly for 6 weeks or until delivery. All women will be asked to deliver at the SMRU unit and data on outcome will be recorded including sex, birthweight, duration of labor, partogram. Dubowitz ‘s score of gestational age and a neonatal neurological examination will be assessed -within 24 hours- in each baby by trained Karen staff. Infants will be examined at birth for the presence of any congenital abnormalities and followed at monthly intervals during the first year of life (**Table ii**) and assessed for their motor and neurological developments, according to the method developed at this site and previously described^16,17^.
- **Sampling at delivery (Table iii):** As for all women with a documented episode of malaria during pregnancy seen in the ANC, blood samples will be obtained from the cord (Haematocrit, malaria smear, PCR), the placenta (crushed smears, biopsies, PCR), the mother and the baby (malaria smear, PCR and haematocrit) on blood obtained by finger stick. In addition a placental biopsy will be obtained and fixed on site in Streck. The women will be encouraged to deliver under supervision in SMRU delivery facilities in Maela Camp. Complicated labour will be transferred to Mae Sot Hospital.

1. **Endpoints**

The primary endpoint of the study will be the PCR adjusted parasitological cure, at day 42 or at delivery depending on which occurs last i.e. if the woman delivers 3 weeks after the treatment, the follow up will continue for another 3 weeks after the delivery. In case of re-appearance of parasites within the follow up period, the genotype will be determined and compared to that of the baseline parasite. In cases of PCR confirmed recrudescence, the plasma level of lumefantrine and the *in vitro* sensitivity of the isolates will be analysed to determine whether the treatment failure is due to a truly resistant parasite and/or a inadequate lumefantrine blood level. The patient will be treated with oral artesunate (12 mg/kg over 7 days) combined to clindamycin (5mg/kg 3 times per day for 7 days), in uncomplicated infections and with IM artemether 3.2 mg/kg or IV artesunate 2.4 mg/kg in case of severe infection. Secondary endpoints include: symptoms clearance, rates of adverse events, biochemistry and haematological, and electrocardiographic anomalies. Other endpoints include: gametocyte carriage, pharmacokinetic parameters including the plasma lumefantrine levels at day 7 as a marker of absorption as well as the infant development during the first year of life^15^ and the histo-pathology examination (presence of parasites, pigments, monocytes infiltrations and other placental changes) of the placenta. The proportions with low birth weight, premature birth, congenital abnormality, stillbirths, neonatal and infant mortality will also be described, although the sample size is insufficient to show a difference in these outcomes.

# Ethical considerations

The proposal has been submitted for approval to the WHO Secretariat Committee on Research Involving Human Subjects. Approval from this Committee will permit the recruitment of the first 20 patients into the Coartem arm. Continuation of the study will depend upon a positive report from the Data Safety Monitoring Committee (DSMC), and submission of the report of the DSMC to the WHO Secretariat Committee on Research Involving Human Subjects. The evaluation of the PK data is not essential to the continuation of the study.

The proposal has been approved (Appendix 8) by the Ethical Committee of the Faculty of Tropical Medicine, Mahidol University in Bangkok. Ethical approval was also granted by the OXTREC (Oxford Tropical Research Ethic Committee, OXTREC # 033-02 approved on the 07^th^ Feb 2003). Each women will be explained in her own language the aims and procedures of the study, the anticipated benefits, and potential inconveniences and hazards of the trial. An explanation form in English, Karen and Burmese languages is attached (Appendix 7). Written consent will be obtained from every patient participating in the study. If the patient is unable to read, an impartial witness will be present for the entire informed consent discussion. After the written informed consent form is read and explained to the patient, and after the patient has orally consented to participation in the trial, and, if capable of doing so, has signed and personally dated the informed consent form, the witness should sign and personally date the consent form. The informed consent will be available in the local language and in English and a signed and dated copy will be supplied to the patient. It will be clearly explained to the patient that she can withdraw from the study at any time without adverse consequences.

# Analysis: statistical and pharmacokinetic

A detailed plan will be finalised before initiation of analyses. We will conduct an intent to treat analysis as well as a per-protocol analysis. The data of the pharmacokinetic study will be analysed using a population PK modelling using the programme WINNONLIN and the derived parameters will be tabulated. The number of women studied should also allow detection of an influence of the trimester on PK/PD parameters. Assays will be performed for lumefantrine and its metabolites according to the method described previously. Samples will be frozen on site in liquid nitrogen and transported on dry-ice.

# Timelines:

# From the start of the trial (month 0, M0) the eligible women will be invited to participate to the pk study. However it is expected that some patients will refuse the sampling or will be too close to delivery to participate to the pk component. Therefore some patients will be recruited in the efficacy study but not in the pk study. After the first 10 women treated with coartem (whether in the pk or not) have delivered, a report will be addressed to the Data Safety Monitoring Committee (DSMC) and to WHO for review. Based on the conclusions of the DSMC, the trial will continue, or be interrupted or the protocol may be subject to amendments. The recruitment in the pk phase is expected to last till M6, the first 10 women are expected to have delivered by M5 and the trial could last between 18 and 24 months.

# External monitoring

The results of the study (focussing on adverse events and pregnancy outcomes) will be subject to an interim analysis of the first 10 patients by a Data Safety Monitoring Committee (DSMC) composed of an obstetrician (Dr Songyot Tangkitchot, Mae Sot), a statistician (Julie Simpson) and an expert in the treatment of malaria in pregnancy (Bernard Brabin, Liverpool). Provided that the committee’s interim report shows no evidence of drug related toxicity to the mother or the foetus that out-weights the benefits of the treatment, another 10 patients will be included and their records reviewed by the DSMC.

After the first 20 patients have been recruited into the Coartem arm and provided the Committee gives it clearance following a review of the data, a protocol amendment will be presented the ethical committees to extend the inclusion criteria to all pregnant women in the second and third trimesters, irrespective of previous treatment with quinine. The evaluation of the PK data is not essential to the continuation of the study. Providing ethical clearance from the ethical committees is obtained, the study will proceed. Every 20 deliveries the DSMC will review the data and the study will go ahead only if the committee gives it approval.

# Data Management

- **Case Record Form (CRF):** Patient information will be collected in the study case record form (Appendix 3). The CRF must be completed legibly for each patient enrolled in the study, in black ink and signed by the Principal Investigator or authorised trial personnel. Patient records will be kept such that the identity of the patient will not be disclosed, and will be available for inspection by the clinical monitors, the Study Director and regulatory authorities. A separate CRF will be completed for the women in the formal pk study (Appendix 4)
- **Data entry:** Data from the CRFs will be entered in a electronic form by two assigned data entry clerks. Specially designed computer checks will be used to identify data errors. Data will be double entered and databases cross-checked for consistency. This method was used by SMRU for the WRAIR audited SPf66 malaria vaccine trial.
- **Quality assurance:** TDR/WHO as the sponsoring agency will perform monitoring visits and subject data recorded in the CRFs to source data verification. Quality Assurance on malaria smears will be performed according to SMRU Standard Operating Procedures (see Appendix 6).

# Administrative Issues

# Good Clinical Practices: This study will adhere to the standards established in the ICH Harmonised Tripartite Guideline for Good Clinical Practices (1 May 1996) and conform to the WHO/TDR Standard Operating Procedures.

# Insurance: The Principal Investigator will be responsible for obtaining appropriate insurance coverage for the study conduct and for taking out separate insurance to be detailed in the budget to WHO/TDR for patient coverage for adverse events related to the study medication.

# Serious and Unexpected AE Reporting: Serious and/or unexpected AEs will be reported immediately or not later than 24 hours by telephone, email or by facsimile to WHO/TDR. The telephone report should be followed by a written report that should fully document the event. Additional laboratory information and clinical data should be supplied as soon as possible, not exceeding 5 days following the event. In case of death, if an autopsy is performed, a copy of the pathological report should be sent to the Sponsor. For each adverse event, even if not serious, the following information must be entered in the Case Record Form: description of adverse event, onset date and type (sudden, gradual, unknown) duration, severity (NCI Common Toxicity Criteria, Appendix 5), cause-effect relationship with the drug (according to the modified Karch and Lasagna's criteria listed in the WHO Standard Operating Procedures for Clinical Investigators), outcome, measures taken (symptomatic treatment, discontinuation of treatment). WHO/TDR will notify the Ministry of Health of Thailand of each SAE reported.

# Archiving: The Principal Investigator will prepare and maintain a file containing the essential documents for the conduct of a clinical trial as listed in the ICH Guidelines for Good Clinical Practices.

# Patient records will be kept such that the identity of the patients will not be disclosed, and will be available for inspection by the clinical monitor and Study Director. CRFs of each study subject will be archived for 3 years. Arrangements will be done by the Sponsor so as to archive them for a longer period, as necessary. Direct access to source data/documents relating to the trial should be granted to the sponsor for trial-related monitoring, audits, Ethical Committee review and regulatory inspections.

# Reports and publications: It is agreed that publication of this study will be permitted. It is understood that if possible there will be, prior to the submission of the manuscript, agreement on the data and their interpretation with the Study Directors.

# Protocol Amendments: After the protocol has been signed, no changes may be made without the agreement of the PI, the Study Directors and Sponsor. Any change will be signed and dated by all parties, attached to the original protocol and submitted for Ethical Committee review.

**References:**

1. Brockman A, Price RN, van Vugt M, et al. Plasmodium falciparum antimalarial drug susceptibility on the north- western border of Thailand during five years of extensive use of artesunate-mefloquine. *Trans R Soc Trop Med Hyg* 2000;**94**(5)**:**537-44.

2. Nosten F, ter Kuile F, Maelankirri L, Decludt B, White NJ. Malaria during pregnancy in an area of unstable endemicity. *Trans R Soc Trop Med Hyg* 1991;**85**(4)**:**424-9.

3. Nosten F, van Vugt M, Price R, et al. Effects of artesunate-mefloquine combination on incidence of Plasmodium falciparum malaria and mefloquine resistance in western Thailand: a prospective study. *Lancet* 2000;**356:**297-302.

4. Nosten F, Vincenti M, Simpson J, et al. The effects of mefloquine treatment in pregnancy. *Clinical Infectious Diseases* 1999;**28**(4)**:**808-15.

5. McGready R, Cho T, Hkirijaroen L, et al. Quinine and mefloquine in the treatment of multidrug-resistant Plasmodium falciparum malaria in pregnancy. *Annals of Tropical Medicine & Parasitology* 1998;**92**(6)**:**643-53.

6. McGready R, Cho T, Cho JJ, et al. Artemisinin derivatives in the treatment of falciparum malaria in pregnancy. *Trans R Soc Trop Med Hyg* 1998;**92**(4)**:**430-3.

7. McGready R, Cho T, Keo NK, et al. Artemisinin antimalarials in pregnancy: a prospective treatment study of 539 episodes of multidrug-resistant Plasmodium falciparum. *Clinical Infectious Diseases* 2001;**33**(12)**:**2009-16.

8. van Vugt M, Ezzet F, Phaipun L, Nosten F, White NJ. The relationship between capillary and venous concentrations of the antimalarial drug lumefantrine (benflumetol). *Trans R Soc Trop Med Hyg* 1998;**92**(5)**:**564-5.

9. van Vugt M, Ezzet F, Nosten F, et al. No evidence of cardiotoxicity during antimalarial treatment with artemether-lumefantrine. *American Journal of Tropical Medicine and Hygiene* 1999;**61**(6)**:**964-7.

10. van Vugt M, Looareesuwan S, Wilairatana P, et al. Artemether-lumefantrine for the treatment of multidrug-resistant falciparum malaria. *Trans R Soc Trop Med Hyg* 2000;**94**(5)**:**545-8.

11. Ezzet F, van Vugt M, Nosten F, Looareesuwan S, White NJ. Pharmacokinetics and pharmacodynamics of lumefantrine (benflumetol) in acute falciparum malaria. *Antimicrobial Agents and Chemotherapy* 2000;**44**(3)**:**697-704.

12. van Vugt M, Brockman A, Gemperli B, et al. Randomized comparison of artemether-benflumetol and artesunate- mefloquine in treatment of multidrug-resistant falciparum malaria. *Antimicrobial Agents and Chemotherapy* 1998;**42**(1)**:**135-9.

13. Assessment of the safety of artemisinin compounds in pregnancy: World Health Organization, 2003.

14. Brockman A, Paul RE, Anderson TJ, et al. Application of genetic markers to the identification of recrudescent Plasmodium falciparum infections on the northwestern border of Thailand. *American Journal of Tropical Medicine and Hygiene* 1999;**60**(1)**:**14-21.

15. Haatja L, McGready R, Arunjerdja R, Simpson JA, Mercuri E, Nosten F, Dubowitz L. A new approach for neurological evaluation of infants in resource-poor settings. *Annals of Tropical Paediatrics* **2002**;22(4):355-68.

pregnant women, Pf positive, in 2^nd^ or 3^rd^ trimester

Screening

randomised

Artesunate, 7 days

Written informed consent

Co-artem, 3 days

First 12 consenting women will have detailed PK study

DSMC clearance to proceed to enrol next 10 women into Coartem group

DSMC to conduct interim analysis of results focussing on AEs and pregnancy outcomes of first 10 deliveries

Artesunate, 7 days

Co-artem, 3 days

DSMC to conduct interim analysis of results focussing on AEs and pregnancy outcomes of next 10 deliveries, and report on whether study can begin to enrol pregnant women who have not had prior history with quinine into Co-artem arm

Artesunate, 7 days

Co-artem, 3 days

Every 20 deliveries DSMC to conduct interim analysis of results focussing on AEs and pregnancy outcomes and clear study to proceed to complete enrolment. Total enrolment 250

DSMC clearance to proceed to enrol next 20 women

DSMC clearance to be provided to ethical committees, protocol amendment, and ethical clearance to proceed to be given

Appendix 1

Defining criteria of severe PF malaria (WHO)[[1]](#footnote-2):

1 Cerebral malaria (unrousable coma)

2 Severe anaemia (Ht<20%)

3 Renal failure (<20 ml urine per hour)

4 Pulmonary oedema

5 Hypoglycaemia (plasma glucose<2.2 mmol/L)

6 Circulatory collapse, shock (systolic BP<80 mmHg)

7 Spontaneous bleeding/disseminated intravascular coagulation

8 Repeated generalized convulsions

9 Acidaemia/acidosis (on clinical signs)

10 Malarial haemoglobinuria

**Other manifestations:**

Impaired consciousness but rousable

Cannot eat and drink by herself

Prostration, extreme weakness

## Hyperparasitaemia

Jaundice

Hyperpyrexia

## Pigment in neutrophils

**Appendix 2 – Drug Dosing Tables**

| **Table iv. Oral Artesunate 2 mg/kg** | |
| --- | --- |
| **(1 Tablet contains 50 mg)** | |
|  |  |
| **Weight (kg)** | **Number of Tablets** |
| **24 - 26** | **1** |
| **27 - 28** | **1** |
| **29** | **1 1/4** |
| **30 - 32** | **1 1/4** |
| **33 - 34** | **1 1/4** |
| **35** | **1 1/2** |
| **36 - 39** | **1 1/2** |
| **40** | **1 1/2** |
| **41 - 42** | **1 3/4** |
| **43 - 45** | **1 3/4** |
| **46** | **1 3/4** |
| **47 - 48** | **2** |
| **49 - 51** | **2** |
| **52 - 53** | **2** |
| **54** | **2 1/4** |
| **55 - 57** | **2 1/4** |
| **58 - 59** | **2 1/4** |
| **60** | **2 1/2** |
| **61 - 64** | **2 1/2** |
| **65** | **2 1/2** |
| **66 - 67** | **2 3/4** |
| **68 - 70** | **2 3/4** |

| **Table v. Coartem ®**  **Number of tablets per dose** | |
| --- | --- |
| **Weight (kg)** | **Number of Tablets** |
| **16-25** | **2** |
| **26-35** | **3** |
| **>35** | **4** |

1. Severe falciparum Malaria supplement, WHO, *Transactions of the Royal*

   *Society of Tropical Medicine and Hygiene* (April 2000) 94, supp. 1, [↑](#footnote-ref-2)
